# Hierarchical Tissue-Specific Modeling of Pathology Images Predicts Response in HER2+ Breast Cancer

**DOI:** 10.34133/cbsystems.0554

**Published:** 2026-04-22

**Authors:** Wensheng Cui, Tao Tan, Ming Fan, Lihua Li

**Affiliations:** ^1^ School of Computer Science and Technology, Hangzhou Dianzi University, Hangzhou 310018, China.; ^2^ School of Computer Science and Information Technology, Daqing Normal University, Daqing 163712, China.; ^3^ Institute of Biomedical Engineering and Instrumentation, Hangzhou Dianzi University, Hangzhou 310018, China.; ^4^ Faculty of Applied Sciences, Macao Polytechnic University, Macao 999078, China.

## Abstract

The structural and spatial heterogeneity of the tumor microenvironment in human epidermal growth factor receptor 2-positive (HER2+) breast cancer (HER2+BC) poses major challenges for predicting pathologic complete response (pCR) to neoadjuvant chemotherapy. While immune-related biomarkers from whole-slide images have been widely explored, tissue-level interaction patterns and semantic features remain underexplored, particularly in characterizing structural organization within tissue compartments. To address this, we propose an interpretable hierarchical framework that models the tissue-specific organization of graph-based structural features and deep-learning-derived pCR scores (DLPSs) and integrates clinical variables to enhance pCR prediction. Whole-slide images were segmented into 5 biologically distinct compartments: tumor, stroma, stromal tumor-infiltrating lymphocytes (sTILs), intratumoral tumor-infiltrating lymphocytes (iTILs), and combined tumor-infiltrating lymphocytes (sTILs plus iTILs). Each compartment was modeled as a graph in which tile-level cluster centers served as nodes and their connections as edges. Structural features were computed using social network analysis (SNA) metrics to characterize spatial organization in each tissue compartment. In parallel, DLPSs were generated using a pretrained clustering-constrained multiple-instance learning model as a feature extractor, followed by training tissue-specific multilayer perceptron classifiers. The tissue-specific SNA features, DLPSs, and clinical variables were integrated for prediction. The model was trained on the Yale Response dataset and externally validated using the IMage-based Pathological REgistration and Segmentation Statistics (IMPRESS) HER2+ dataset. Across compartments, stroma achieved the highest predictive performance (area under the receiver operating characteristic curve [AUC] = 0.907), surpassing a reported method by 9.5%. Notably, SNA features achieved an AUC of 0.793, outperforming DLPS (0.596) and clinical variables (0.757). These findings suggest the value of integrating tissue-specific structural and semantic features for interpretable modeling of treatment response variability in HER2+BC.

## Introduction

Breast cancer remains a leading cause of cancer-related morbidity and mortality among women worldwide [1]. Human epidermal growth factor receptor 2-positive breast cancer (HER2+BC) accounts for approximately 20% of breast cancer cases and is associated with aggressive tumor behavior and an increased risk of distant metastasis [2–4]. Among molecular subtypes, HER2+BC exhibits biological characteristics intermediate between hormone receptor-positive and triple-negative breast cancers [5,6]. Neoadjuvant chemotherapy (NAC) is a standard treatment strategy for HER2+BC, and pathologic complete response (pCR) following NAC is a well-established prognostic indicator associated with improved survival outcomes [7–9]. Despite its widespread use, approximately 30% of patients exhibit limited response to NAC [10]. The tumor microenvironment (TME) has emerged as a critical determinant of response to NAC [11,12]. Histopathological images, which remain the diagnostic gold standard, inherently preserve the spatial and structural characteristics of the TME, including compartmental interactions and morphological heterogeneity [13]. Therefore, effectively modeling the spatial and structural information in histopathological images is essential for predicting pCR in HER2+BC.

Deep learning (DL) has markedly advanced computational pathology, enabling the automated interpretation of histopathological images across a variety of tasks [14]. In computational pathology, DL has been widely applied to fundamental histological analysis tasks such as cell segmentation and tissue classification and has more recently been extended to support higher-level histopathological analysis [15,16]. Recent studies have demonstrated that DL models can learn informative representations of the TME directly from routine hematoxylin and eosin (H&E)-stained slides [17–19]. These representations have been shown to capture tissue-level spatial and semantic patterns relevant to clinically important tasks, including disease subtyping and outcome or treatment response prediction [20–23]. Collectively, these advances indicate that DL-based approaches provide a promising foundation for modeling clinically relevant TME heterogeneity in breast cancer.

**Fig. 1.**
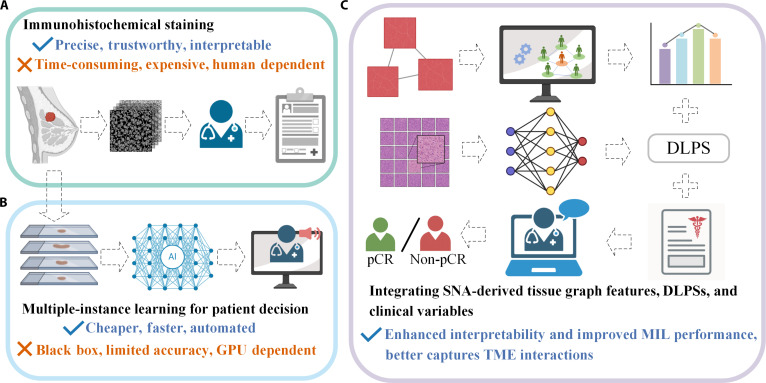
Strategies for pathology-based prediction of pathologic complete response (pCR) to neoadjuvant chemotherapy (NAC) in breast cancer. (A) Immunohistochemical staining is precise and interpretable but time-consuming and labor-intensive. (B) Deep-learning (DL)-based multiple-instance learning enables automated prediction with limited interpretability. (C) Our method integrates DLPSs, spatial graph features, and clinical variables to improve both accuracy and interpretability by modeling whole-slide-level tissue interactions. Check marks indicate methodological advantages, whereas cross marks denote limitations. DLPSs, DL-derived pCR scores.

In current clinical practice, immunohistochemical (IHC) staining is commonly used to assess biomarkers associated with NAC response in breast cancer, such as Ki-67, sperm-associated antigen 5, and programmed death-ligand 1 (Fig. 1A) [24–26]. Although clinically effective, IHC-based approaches are labor-intensive, time-consuming, and dependent on expert interpretation, which limits their scalability in routine workflows [27]. To improve efficiency and generalizability, recent studies have explored DL approaches applied to both IHC- and H&E-stained whole-slide images (WSIs) [28–30]. Representative IHC-based DL studies have demonstrated improved predictive performance compared with manual scoring; however, these approaches often rely on nonroutine staining protocols and remain limited by insufficient external validation and interpretability [31,32].

As H&E slides are routinely available in clinical practice, they have become a preferred modality for scalable DL applications. Li et al. [28,29] developed a pipeline using tumor and stromal compartment segmentation to compute predictive scores, reporting high areas under the receiver operating characteristic curve (AUCs) in external cohorts. More recently, Shen et al. [30] introduced a 3-branch convolutional neural network (CNN) to model TME heterogeneity. Farahmand et al. [33] also applied a multiple-instance learning (MIL)-based method for response prediction in HER2+BC. Despite these advances, most existing approaches rely on weakly supervised learning frameworks such as MIL, which treats WSIs as unordered tiles, thereby limiting explicit modeling of spatial organization [34]. Moreover, although multimodal strategies combining molecular or genomic data have been proposed, such data are rarely available in routine clinical settings, and DL-derived features often lack clear histopathologic interpretability (Fig. 1B) [35–37].

To explicitly model spatial organization within histopathological images, graph-based frameworks have been proposed to encode tissue architecture and spatial relationships among histological components. For instance, Shao et al. [38] constructed tissue graphs based on tumor, stromal, and lymphocyte compartments for survival analysis, while Di et al. [39] developed a hypergraph model to capture higher-order spatial dependencies. More recently, Li et al. [40] introduced NACNet, which constructs tile-level spatial graphs to predict NAC response. However, these methods typically emphasize fine-grained spatial connectivity and often operate without explicit compartment-level biological semantics, which may limit interpretability for pCR prediction. In addition, most existing graph-based frameworks focus on single data modality and rarely integrate deep semantic representations with clinically relevant variables in a unified framework.

Recent studies have highlighted mesoscale heterogeneity within the TME as a key factor influencing pCR in the setting of NAC [41,42]. However, whether different TME compartments provide complementary, tissue-specific signals for pCR prediction under a unified modeling framework remains unclear. Building on this insight, we propose a biologically grounded, tissue-specific modeling framework that operates at the mesoscale to characterize spatial organization within the TME. Specifically, we construct independent tissue graphs for 5 key compartments observed in H&E-stained WSIs, extract interpretable mesoscale structural features using social network analysis (SNA), and integrate these features with DL-derived pCR scores (DLPSs) obtained from a weakly supervised attention-based model, together with clinical variables, for final prediction. By relying solely on routinely available H&E-stained slides, this framework offers a potentially scalable approach compatible with standard H&E workflows (Fig. 1C). The primary contributions of this work are as follows: (a) we introduce a tissue-specific graph modeling framework that constructs independent graphs for 5 biologically meaningful TME compartments and derives interpretable mesoscale structural features using SNA, and (b) we integrate graph-derived structural features with DLPSs from a clustering-constrained multiple-instance learning model (CLAM) and clinical variables to form a hierarchical representation that improves predictive performance and histopathologic interpretability.

## Materials and Methods

### Materials

A total of 147 patients with HER2+BC who received NAC were included in this study from 2 publicly available cohorts. The Yale Response dataset from Yale University (*n* = 85) was used for model development, while the IMage-based Pathological REgistration and Segmentation Statistics (IMPRESS) HER2+ dataset from Purdue University (*n* = 62) served as an external validation cohort to assess model generalizability. Patients were required to be diagnosed with HER2+BC and to have available pretreatment H&E-stained WSIs obtained from core needle biopsies, together with corresponding clinical information. Cases were excluded only if pretreatment WSIs were missing, severely damaged, or of insufficient quality for computational analysis. No additional selection based on treatment response or histopathological characteristics was applied. As a result, no cases were excluded due to missing clinical outcome data, and all 147 patients were retained for subsequent analysis. This study was conducted using publicly available and de-identified datasets. Ethical approval and informed consent were obtained as part of the original studies from which the datasets were derived. The use of human data for this secondary analysis was reviewed and approved by the Institutional Review Board of Hangzhou Dianzi University, in accordance with institutional and regulatory requirements. pCR was defined as the absence of residual invasive carcinoma in the breast and axillary lymph nodes (ypT0/Tis and ypN0), according to routine pathological assessment.

The Yale Response dataset consisted of WSIs derived from pretreatment core needle biopsies, along with corresponding clinical data from 85 female patients diagnosed with HER2+BC [33]. All slides were digitized at 20× magnification using Aperio ScanScope Console (version 10.2.0.2352). Therapeutic response to NAC was evaluated by board-certified pathologists based on the examination of surgical resection specimens, and pCR was determined according to the above definition. The IMPRESS HER2+ dataset included 62 patients with HER2+BC, each represented by H&E-stained WSIs from pretreatment core needle biopsies and associated clinical data [31]. Slides were digitized at 20× magnification using a Hamamatsu scanner. Most patients received NAC regimens containing a taxane, either paclitaxel or docetaxel, in combination with trastuzumab. A subgroup of 7 patients received a 4-cycle regimen comprising pertuzumab, trastuzumab, and docetaxel. Among these, 4 patients achieved pCR and 3 did not. Neoadjuvant treatment regimens primarily consisted of taxane-based chemotherapy combined with anti-HER2 therapy. Pathologic response was evaluated using the same pCR definition as described above.

Clinical and histopathological variables were summarized for descriptive analysis. Categorical variables (e.g., estrogen receptor [ER]/progesterone receptor [PR] status) were analyzed using the chi-square test, while ordinal variables (e.g., Nottingham grade and nuclear grade) and continuous variables were compared using the Mann–Whitney *U* test. An overview of patient characteristics is provided in Table 1.

**Table 1.** Clinical and histopathological characteristics of HER2+ patients in the 2 datasets

Cohort	Characteristics		No. of cases/median	%/range	*P* value
Yale Response dataset	Total case number		85	-	-
	Cases with pCR		36	42.35%	-
	Cases with residual tumor		49	57.65%	-
	ER positive		69	81.18%	0.878
	PR positive		66	77.65%	0.773
	HER2/CEP17 ratio		4.35	0.00–17.40	0.027
	RIS/cm		1.35	0.02–11.00	<0.001
	HER2CN/(signals·cell^−1^)		11	0.00–26.70	0.136
IMPRESS HER2+ dataset	Total case number		62	-	-
	Cases with pCR		38	61.29%	-
	Cases with residual tumor		24	38.71%	-
	ER positive		30	48.39%	0.042
	PR positive		19	30.65%	0.019
	HER2/CEP17 ratio		6.73	1.23–22.98	<0.001
	RIS/cm		0.80	0.10–7.00	<0.001
	Residual cancer burden		1.39	0.91–4.14	<0.001
	Age/years		56	30–76	0.104
	Nottingham grade	I	1	1.61%	0.829
		II	27	43.55%	
		III	34	54.84%	
	Nuclear grade	I	0	0.00%	0.937
		II	10	16.13%	
		III	52	83.87%	

HER2, human epidermal growth factor receptor 2; pCR, pathologic complete response; ER, estrogen receptor; PR, progesterone receptor; RIS, residual infiltration size; CEP17, centromere of chromosome 17; HER2CN, HER2 copy number; IMPRESS, IMage-based Pathological REgistration and Segmentation Statistics; “-” indicates not applicable or not available

### Method overview

**Fig. 2.**
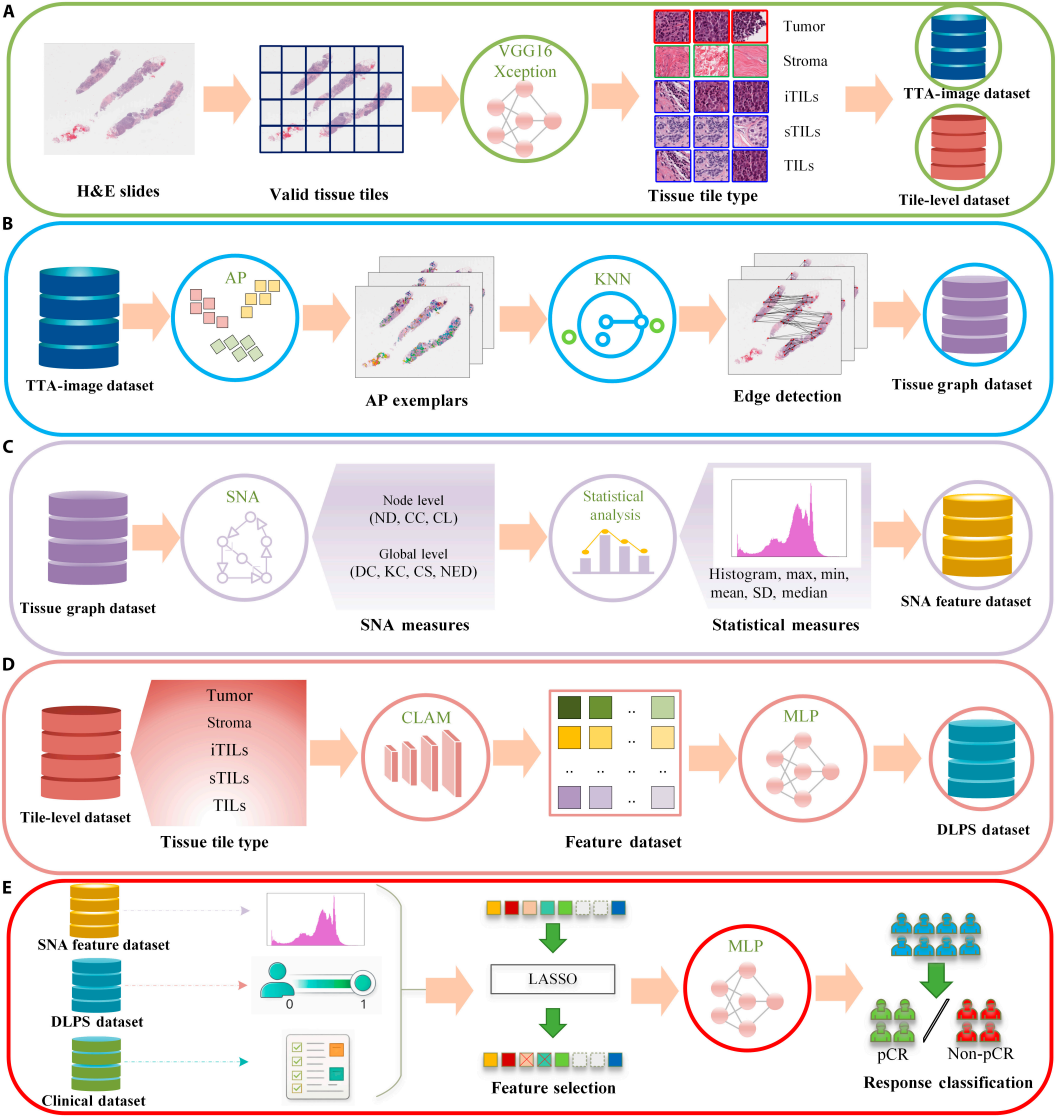
Overview of the proposed framework for predicting pathologic complete response (pCR) to neoadjuvant chemotherapy (NAC) in human epidermal growth factor receptor 2-positive breast cancer (HER2+BC). (A) Generation of tile-level and tissue type annotation image (TTA-image) datasets from hematoxylin and eosin (H&E)-stained whole-slide images (WSIs). (B) Extraction of tissue-specific deep-learning-derived pCR scores (DLPSs) at the patient level using a constrained multiple-instance learning model (CLAM). (C) Construction of tissue-specific graphs, in which cluster exemplars identified by affinity propagation (AP) clustering are selected as graph nodes, and spatial relationships are modeled via *K*-nearest neighbors (KNNs)-based edges. (D) Derivation of interpretable graph features using social network analysis (SNA), followed by statistical summarization at the patient level. (E) Integration of DLPSs, SNA-derived features, and clinical variables, with feature selection via LASSO and final pCR classification using a multilayer perceptron (MLP). LASSO, least absolute shrinkage and selection operator; ND, node degree; CC, closeness centrality; CL, clustering coefficient; DC, degree centrality; KC, Katz centrality; CS, community structure; NED, network density.

Fig. 2 presents the overall framework for predicting pCR to NAC in patients with HER2+BC. The proposed method integrates hierarchical information derived from H&E-stained WSIs, encompassing deep semantic features, graph-based spatial descriptors, and clinical data. The workflow comprises 5 main stages. First, tumor and stroma compartments are identified using tile-level CNN classification and mapped back to WSIs, followed by the identification of stromal tumor-infiltrating lymphocytes (sTILs) and intratumoral tumor-infiltrating lymphocytes (iTILs). These lymphocyte populations are combined to define the overall tumor-infiltrating lymphocyte (TIL) compartment, which is used to generate tile-level datasets and the corresponding tissue type annotation images (TTA-images). Second, DLPSs are independently computed for each tissue compartment by extracting tile-level embeddings from a pretrained CLAM model (used as a fixed feature extractor), followed by training lightweight tissue-specific multilayer perceptron (MLP) classifiers. Third, tissue-specific graphs are constructed from TTA-images via affinity propagation (AP) clustering, where cluster centers define graph nodes and spatial proximity determines the edge structure [43]. Fourth, SNA is applied to extract interpretable structural features from each graph. Finally, features extracted from each tissue compartment, including graph descriptors and DLPSs, are combined with patient-level clinical variables to develop individual predictive models. These models are subsequently integrated into a tissue-specific classifier for pCR prediction.

A modular design was preferred over an end-to-end WSI-level model because it enables explicit decomposition of the pipeline and supports downstream analysis and interpretation, whereas end-to-end approaches are primarily optimized as black-box predictors [13,44]. Although the framework comprises multiple stages, each module serves a distinct and nonoverlapping role, enabling independent inspection and module-level evaluation for quality control. To control pipeline complexity and reduce sensitivity to local variations, spatial information is summarized at the graph level using representative nodes and statistical descriptors. The robustness of the complete pipeline is further evaluated through cross-validation and independent external validation. In the subsequent sections, we describe each step of the pipeline in detail.

### Generation of tile-level data and TTA-images

**Fig. 3.**
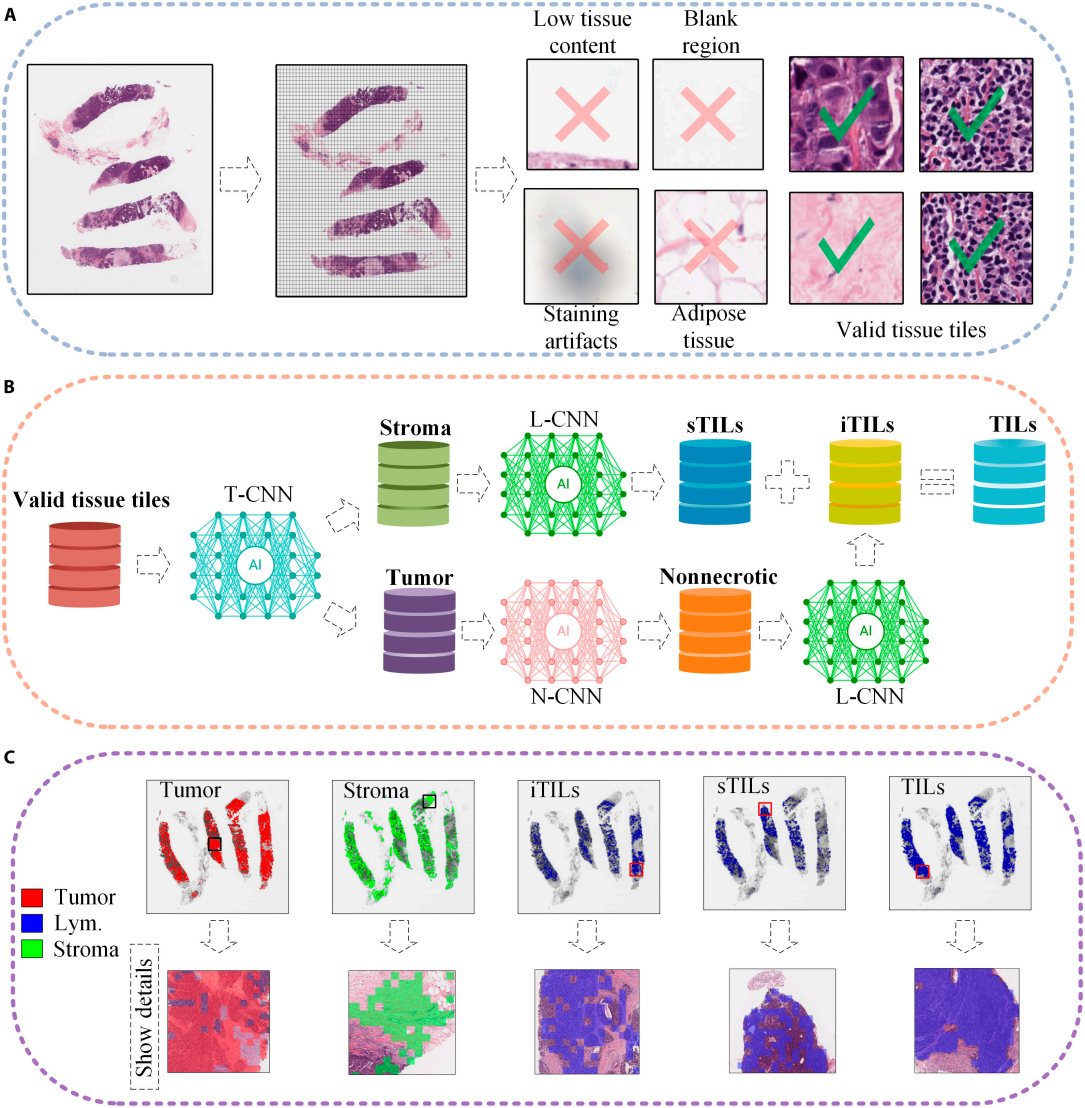
Pipeline for generating tile-level and tissue type annotation image (TTA-image) datasets. (A) Hematoxylin and eosin (H&E)-stained whole-slide images (WSIs) are divided into nonoverlapping tiles. Tiles corresponding to low tissue content, blank regions, staining artifacts, and adipose tissue are excluded, while tiles containing valid tissue structures are retained for downstream analysis. (B) A sequential convolutional neural network (CNN)-based framework is applied to classify valid tissue tiles into tumor, stroma, necrotic, and lymphocyte-infiltrated compartments using task-specific CNNs. (C) Visualization of TTA-images for a representative human epidermal growth factor receptor 2-positive breast cancer (HER2+BC) case from the IMPRESS dataset (patient ID: 080_HE), illustrating tissue-specific spatial distributions, including tumor, stroma, intratumoral tumor-infiltrating lymphocytes (iTILs), stromal tumor-infiltrating lymphocytes (sTILs), and tumor-infiltrating lymphocytes (TILs). T-CNN, CNN for tumor classification; N-CNN, CNN for necrosis classification; L-CNN, CNN for lymphocyte-infiltration classification; Lym., lymphocytes; IMPRESS, IMage-based Pathological REgistration and Segmentation Statistics.

To prepare data for downstream modeling, WSIs were processed to generate both tile-level datasets and TTA-images. The preprocessing pipeline consists of 3 main steps, namely, tile extraction and filtering, tissue classification, and TTA-image reconstruction, as illustrated in Fig. 3.

#### Tile extraction and preprocessing

WSIs were partitioned into nonoverlapping square tiles measuring 87.5 μm per side (175 pixel × 175 pixel) at 20× magnification (0.5 μm/pixel) using the OpenSlide toolkit [45]. This tile size was chosen to balance the preservation of mesoscale histological structures with computational efficiency, enabling the capture of local tissue architecture while avoiding excessive fragmentation. Images acquired at different magnifications were rescaled to a common spatial resolution to ensure consistent tile representation across datasets. To remove low-informative compartments, tissue regions were first segmented using Otsu’s thresholding, and the proportion of tissue pixels within each tile was calculated. Tiles containing less than 20% tissue area were discarded. In addition, tiles corresponding to blank regions, staining artifacts, and adipose tissue were excluded, retaining only diagnostically relevant tissue tiles for downstream analysis.

#### Tissue classification and TTA-image generation

To enable tissue-level analysis, CNNs were trained to identify tumor, lymphocyte, and necrotic compartments using a transfer learning framework [46]. Tile-level predictions were treated as discrete tissue labels and mapped back to their corresponding spatial coordinates on the WSI. This procedure reconstructed a 2-dimensional representation of tissue distribution, referred to as TTA-images, which served as the structural foundation for graph construction and spatial analysis. A TTA-image comprises 5 biologically relevant compartments. Tumor and stroma compartments were identified using tile-level CNN classification and mapped back onto WSIs. iTILs and sTILs were then identified based on the spatial localization of lymphocytes within these respective areas. The overall TIL compartment is subsequently defined by combining iTILs and sTILs. Compared to pixel-level or tile-level predictions, TTA-images provide a structured and spatially coherent representation that is well suited for downstream graph construction. We adopted a tile-based tissue classification strategy rather than pixel-level segmentation models (e.g., U-Net), because the proposed framework focuses on tissue-scale and mesoscale spatial organization, for which region-level representations are sufficient.

In this study, the TME was defined as the combined area of tumor and its adjacent stroma, excluding adipose tissue, necrotic zones, ink artifacts, and blank compartments removed during preprocessing. Tissue classification was performed on H&E-stained core needle biopsy slides using DL models trained on pathologist-annotated compartments, enabling reproducible delineation of TME compartments that is consistent with commonly used histopathologic definitions. The stromal compartment denotes nonmalignant supportive tissue within the TME. sTILs and iTILs are defined as lymphocytes located in stromal regions and infiltrating tumor compartments, respectively.

Due to the imbalanced sample sizes and class distributions across tissue types, a single multiclass model may be suboptimal. To address this issue, we adopted a sequential binary classification strategy, as illustrated in Fig. 3B. The first step involved distinguishing tumor compartments from stromal compartments. Given the morphological similarity between necrotic tissue and iTILs, a separate CNN was trained to identify necrosis and minimize false positives [47]. iTILs were then classified, followed by the identification of sTILs from the remaining stromal compartments. Finally, iTILs and sTILs were combined to form TILs.

In practice, tumor–stroma classification was performed using a fine-tuned Xception model, whereas necrosis and lymphocyte classification relied on fine-tuned VGG-16 models, all trained using cross-entropy loss under a transfer learning framework. The tissue classification models demonstrated stable and consistent performance across major tissue compartments. Specifically, tumor–stroma classification achieved an accuracy of 0.865 on the validation set and 0.860 on the independent test set, with corresponding AUC values of 0.927 and 0.917, respectively. Necrosis classification achieved a mean accuracy of 0.964 ± 0.035 under stratified 5-fold cross-validation, while lymphocyte classification achieved a mean accuracy of 0.914 ± 0.031 under the same evaluation protocol. Because downstream graph construction is based on AP-clustered exemplar nodes and graph-level SNA features that characterize mesoscale spatial organization within each tissue compartment, the proposed framework is less sensitive to isolated tile-level misclassifications. These results suggest that the generated TTA-images provide reasonably reliable upstream inputs for subsequent graph construction and spatial analysis. Further details regarding the CNN architectures, datasets, training procedures, and evaluation results are provided in Section S1 of Data File S1.

### Tissue network generation

**Fig. 4.**
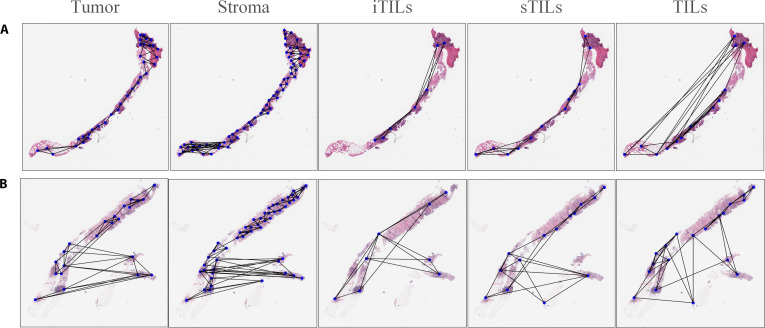
Tissue networks of tumor, stroma, intratumoral tumor-infiltrating lymphocytes (iTILs), stromal tumor-infiltrating lymphocytes (sTILs), and tumor-infiltrating lymphocytes (TILs) from representative cases in the Yale Response dataset. Panels (A) and (B) correspond to patients S12-07776 (pathologic complete response [pCR]) and S18-09519 (non-pCR), respectively. Each blue node represents a tissue cluster generated by affinity propagation (AP), and black edges denote spatial proximity defined by *K*-nearest neighbor (KNN). These networks illustrate intracompartment spatial organization across different tissue compartments.

To capture the spatial distribution of different tissue compartments, 5 independent graphs were constructed from the TTA-images, corresponding to tumor, stroma, iTILs, sTILs, and TILs. Representative tissue networks are shown in Fig. 4. These graphs encode the spatial architecture of tumor and immune components within the TME and provide interpretable features for pCR prediction. In each graph, denoted as G = (V, E), nodes represent localized tissue compartments and edges indicate their spatial proximity.

The AP algorithm was used to group tissue tiles, with the resulting exemplars (cluster representatives) treated as graph nodes. The AP preference values do not explicitly specify the number of clusters but implicitly control exemplar selection and clustering granularity, with more negative values yielding fewer exemplars and coarser spatial representations. Compared to assigning a node to every tile, this strategy reduces noise and computational burden while preserving the spatial organization of biologically meaningful compartments. Edges were established by connecting each node to its *K*-nearest neighbors (KNNs) based on Euclidean distance [48]. We evaluated the influence of the AP preference value (*P*), which controls clustering granularity, and the KNN parameter (*K*), which controls local graph connectivity, on graph construction and predictive performance. A grid-based evaluation was conducted on the Yale Response dataset using a discrete set of AP preference values (*P* = −100, −50, and −20) and KNN neighborhood sizes (*K* = 4, 5, and 6), under stratified 5-fold cross-validation, followed by validation on the independent IMPRESS HER2+ dataset. Across these parameter settings, different tissue compartments exhibited distinct clustering granularities, consistent with their spatial continuity, and sensitivity analyses demonstrated stable graph-derived feature performance across both cross-validation and external validation. For transparency, we report the number of AP-generated graph nodes for each patient and each tissue compartment, together with summary statistics by tissue type, in Data File S5. Detailed parameter evaluations and performance metrics are provided in Section S2 of Data File S1.

Graph connectivity can capture spatial patterns within the TME. For example, in TIL-related graphs, higher connectivity may correspond to more localized lymphocyte clustering, whereas sparse connectivity may correspond to a more dispersed distribution. Similarly, in tumor or stromal graphs, denser networks may correspond to more compact tissue regions, while sparser networks may correspond to greater spatial dispersion or heterogeneity. These patterns are quantified using graph-theoretic metrics (e.g., average degree) and subsequently used as predictive features. To facilitate qualitative understanding, we present representative pCR and non-pCR cases to visually illustrate these spatial differences.

### SNA-based feature extraction

Based on the constructed tissue-specific graphs, structural features were extracted using SNA to capture the spatial characteristics of tissue organization within the TME. These features are designed to be low-dimensional, interpretable, and complementary to DL representations, providing additional insights into tissue-level spatial patterns [49,50]. Importantly, our framework does not employ graph neural networks; instead, we rely on handcrafted graph-theoretic descriptors derived from SNA and subsequently modeled using a shallow MLP, which facilitates interpretability by enabling direct attribution of predictive patterns to explicit structural metrics. Seven SNA metrics were selected to capture diverse aspects of graph structure, including node degree (ND), clustering coefficient (CL), closeness centrality (CC), degree centrality, Katz centrality, network density (NED), and community structure (CS) [51]. Each metric reflects a distinct structural property of the graph and may have potential biological relevance. Formal definitions and mathematical formulations of all SNA metrics are provided in Section S3 of Data File S1, with corresponding feature names listed in Data File S2. Feature abbreviations in subsequent figures follow this nomenclature and are not repeated in the main text for brevity.

To convert the node-level SNA features into input suitable for modeling, a statistical summarization strategy was employed. For each of the 6 node-level SNA metrics, we computed a 10-bin histogram along with 5 summary statistics, namely, the mean, standard deviation, maximum, minimum, and median, across all nodes within a graph. This yielded 15 features per metric and resulted in a total of 90 features for each graph. NED, as a graph-level metric, was appended directly, producing a 91-dimensional feature vector for each tissue graph. This approach is computationally efficient, does not rely on precise pixel-level annotations, and is robust to noise. It enables structural relationships among tissue compartments to be quantitatively represented and easily integrated with other feature types. The complete extraction process is illustrated in Fig. 2C.

### DLPS generation

To extract WSI-level semantic features under weak supervision, we employed the CLAM model, which enables whole-slide classification using only slide-level labels [52]. In CLAM, each slide is modeled as a collection of tiles, and informative instances are identified through attention-based pooling constrained by instance-level clustering. A pretrained CLAM encoder, originally trained on large-scale histopathology datasets covering diverse cancer types, was used as a frozen feature extractor to generate 1,024-dimensional tile-level embeddings. These embeddings were subsequently used for downstream prediction of pCR. We chose to freeze the encoder weights during downstream training to mitigate overfitting and ensure stable feature representations, given the limited size of our cohort.

To estimate treatment response at the WSI level, a lightweight MLP was trained to classify pCR status based on the tile representations. The model output probability scores for individual tiles, which were then summarized within each tissue compartment by calculating the mean and standard deviation. This procedure was applied independently to tumor, stroma, iTILs, sTILs, and their union TILs, resulting in 10 response-related features. These DLPSs were integrated with graph-derived spatial descriptors and clinical variables to inform the final predictive model. For reproducibility, the downstream MLP was trained using the Adam optimizer (learning rate = 0.0001 and batch size = 512) with dropout regularization on the Yale Response dataset using a stratified 80/20 patient-level split for up to 500 epochs, with early stopping based on validation loss; full architectural details and training settings are provided in Section S4 of Data File S1.

### Hierarchical feature integration and tissue-specific prediction

To investigate the predictive value of different tissue compartments in HER2+BC, a tissue-specific modeling strategy was employed. Rather than aggregating all features into a single unified representation, separate MLPs were trained for each of the 5 tissue compartments: tumor, stroma, iTILs, sTILs, and TILs. For each compartment, a 99-dimensional input vector was constructed, comprising 91 graph-based features extracted through SNA, 2 DL-derived scores representing the mean and standard deviation of DLPS within the compartment, and 6 shared clinical variables, including ER and PR status and percentage, centromere of chromosome 17 (CEP17), and the HER2/CEP17 ratio, as illustrated in Fig. 2E.

Clinical variables were appended to each tissue-specific model as shared baseline covariates, so that the incremental value of compartment-derived (SNA/DLPS) features could be evaluated under the same clinical context rather than being confounded by differences in clinical case mix across compartments. Each compartment was modeled independently, with its corresponding features used to predict pCR using a dedicated classifier. Importantly, features from different tissue compartments were not combined into a single multicompartment model, in order to avoid cross-compartment collinearity and to preserve the interpretability of tissue-specific effects. To reduce redundancy and enhance model generalization, feature selection was performed using the least absolute shrinkage and selection operator (LASSO). Given the limited dataset size, an MLP was chosen as the predictive model due to its demonstrated effectiveness in handling low-dimensional tabular data. Hyperparameters for LASSO were optimized using cross-validation, while those of the downstream MLP classifier for final pCR prediction were selected via grid search with stratified 5-fold cross-validation using the standard scikit-learn implementation.

Unless otherwise specified, a fixed random seed was used to ensure reproducibility, and model selection and hyperparameter tuning were performed using stratified 5-fold cross-validation on the Yale Response dataset. For individual clinical variables (e.g., ER, PR, and related quantitative measures), variables were numerically encoded where applicable and independently used as the sole input to train lightweight predictive models, from which continuous prediction scores were generated to compute AUC values.

### Evaluation metrics and statistical analysis

To compare feature distributions between the pCR and non-pCR groups, categorical variables were analyzed using the chi-square test, while ordinal and continuous variables derived from SNA- and DL-based features were compared using the Mann–Whitney *U* test within both the Yale Response and IMPRESS HER2+ datasets. To account for multiple comparisons, *P* values were adjusted using the Benjamini–Hochberg false discovery rate (BH-FDR) procedure, and BH-FDR-adjusted *P* values were used to determine statistical significance. Furthermore, Spearman’s rank correlation coefficients were computed to assess the associations between extracted features and the residual infiltration size (RIS), defined as the longest diameter of the remaining invasive lesion after NAC. Each correlation was summarized by its Spearman coefficient (*ρ*) and the corresponding BH-FDR-adjusted *P* value.

To evaluate model performance, we report the AUC, F1 score, positive predictive value, recall, and negative predictive value. AUC comparisons across different models and feature sets were performed using the DeLong test. When assessing results on the independent validation cohort, an AUC greater than 0.800 is commonly interpreted as indicative of strong discrimination, whereas an AUC exceeding 0.700 is commonly interpreted as suggestive of potentially useful classification capability.

### Computational environment

All experiments were conducted on a high-performance computing cluster equipped with 2 NVIDIA Quadro RTX 6000 graphics processing units and 2.0 TB of local storage. The software environment included OpenSlide (v1.2.0) for WSI tiling and TensorFlow (v2.4.1) for DL model implementation. All machine learning and statistical analyses were carried out in Python using scikit-learn (v1.2.2) and SciPy (v1.8.1). Image-level feature extraction and data preprocessing were performed using OpenCV (v4.7.0), Pandas (v2.0.2), and scikit-image (v0.19.3).

## Results

### Sensitivity analysis of graph construction parameters

To avoid confounding effects from DL-based or clinical features, we performed parameter sensitivity analysis using SNA features alone. We investigated the impact of graph construction parameters on SNA feature extraction and performance by varying the number of nearest neighbors (*K* = 4, 5, and 6) and AP preference values (*P* = −100, −50, and −20). As shown in Section S2 of Data File S1, performance was evaluated through stratified 5-fold cross-validation on the Yale Response dataset across all 5 tissue compartments. Across all parameter combinations, the configuration of *K* = 4 and *P* = −50 consistently achieved the highest or near-highest performance, with the tumor and TIL compartments exhibiting particularly strong discriminative ability (average AUCs of 0.819 and 0.812, respectively). The robustness of this configuration was further confirmed on the IMPRESS HER2+ external validation dataset (see Section S2 of Data File S1), where similar performance trends were observed. These results suggest the robustness and discriminative capacity of SNA-derived graph features under this configuration, which was adopted in all subsequent modeling steps. This highlights that careful selection of graph parameters is important for stable spatial modeling and downstream predictive performance.

### Hierarchical tissue-specific modeling for pCR prediction

We evaluated the predictive performance of different feature combinations across 5 tissue compartments, including DLPS, SNA-derived features, and clinical variables. As presented in Table 2 and Section S5 of Data File S1, the integration of all 3 feature types consistently yielded the highest AUCs across most compartments.

**Table 2.** Model performance of different feature combinations across 5 tissue compartments on the IMPRESS HER2+ external validation dataset. For each tissue compartment, the best-performing feature combination is highlighted in bold based on AUC.

Feature type	Compartment	AUC	F1 score	PPV	Recall	NPV
SNA + DLPS + clinical	Tumor	**0.764**	0.828	0.735	0.947	0.800
SNA + DLPS		0.720	0.800	0.692	0.947	0.750
SNA + clinical		0.739	0.779	0.769	0.789	0.640
DLPS + clinical		0.669	0.795	0.700	0.921	0.714
SNA		0.654	0.750	0.621	0.947	0.500
DLPS		0.620	0.769	0.660	0.921	0.636
SNA + DLPS + clinical	Stroma	**0.907**	0.892	0.889	0.895	0.792
SNA + DLPS		0.799	0.800	0.811	0.789	0.667
SNA + clinical		0.879	0.853	0.865	0.842	0.741
DLPS + clinical		0.815	0.786	0.717	0.868	0.667
SNA		0.793	0.795	0.700	0.921	0.714
DLPS		0.596	0.750	0.660	0.868	0.571
SNA + DLPS + clinical	iTILs	0.750	0.805	0.673	1.000	0.833
SNA + DLPS		0.561	0.657	0.657	0.657	0.435
SNA + clinical		0.660	0.780	0.681	0.914	0.636
DLPS + clinical		**0.769**	0.805	0.750	0.868	0.700
SNA		0.649	0.759	0.732	0.789	0.591
DLPS		0.541	0.768	0.623	1.000	0.667
SNA + DLPS + clinical	sTILs	0.818	0.833	0.811	0.857	0.714
SNA + DLPS		0.797	0.831	0.762	0.914	0.750
SNA + clinical		0.754	0.800	0.800	0.800	0.652
DLPS + clinical		**0.838**	0.840	0.773	0.919	0.778
SNA		0.669	0.737	0.683	0.800	0.529
DLPS		0.647	0.744	0.653	0.865	0.538
SNA + DLPS + clinical	TILs	**0.803**	0.857	0.783	0.947	0.812
SNA + DLPS		0.644	0.722	0.765	0.684	0.536
SNA + clinical		0.788	0.833	0.761	0.921	0.750
DLPS + clinical		0.616	0.781	0.665	0.947	0.667
SNA		0.667	0.776	0.633	1.000	0.500
DLPS		0.565	0.760	0.613	1.000	0.500
Clinical	-	0.757	0.771	0.711	0.842	0.632

HER2, human epidermal growth factor receptor 2; AUC, area under the receiver operating characteristic curve; PPV, positive predictive value; NPV, negative predictive value; SNA, social network analysis; DLPS, deep-learning-derived pathologic complete response score; iTILs, intratumoral tumor-infiltrating lymphocytes; sTILs, stromal tumor-infiltrating lymphocytes; TILs, tumor-infiltrating lymphocytes; IMPRESS, IMage-based Pathological REgistration and Segmentation Statistics; “-” indicates not applicable or not available

The stromal compartment exhibited the highest overall predictive performance, achieving an AUC of 0.907, an F1 score of 0.892, and a recall of 0.895, indicating that tissue regions beyond the tumor itself can carry strong predictive signals for pCR. In immune-related compartments, incorporating DLPS consistently improved model performance. Specifically, the combination of DLPS and clinical features achieved an AUC of 0.838 in the sTIL compartment, while the addition of DLPS increased the AUC to 0.769 in the iTIL compartment, compared with models using SNA features or clinical variables alone. Across compartments, SNA features demonstrated strong standalone predictive value and outperformed DLPS alone in several settings, particularly in the stromal and TIL compartments. Clinical features further contributed to performance improvements across all tissue compartments, highlighting the importance of biological context. Overall, these results support the effectiveness of the tissue-specific modeling strategy and underscore the value of integrating structural, semantic, and clinical information for accurate pCR prediction.

### Comparative evaluation against baselines and prior work

To further evaluate the predictive performance of our framework, we compared it against a range of clinical baselines and previously published methods, as summarized in Table 3. The comparison set includes commonly used clinical markers, such as ER and PR status and the HER2/CEP17 ratio, as well as handcrafted features derived from tile-level counts of tumor, stroma, and TILs. In addition, we assessed models that incorporate relative ratios between tissue compartments, including the tumor-to-stroma ratio, with formal definitions provided in Section S6 of Data File S1.

**Table 3.** Comparison of model performance with other recent studies on the IMPRESS HER2+ external validation dataset. Bold indicates the highest AUC among all compared methods on the IMPRESS HER2+ external validation dataset.

Type	Method	AUC	F1 score	PPV	Recall	NPV
Our method	Tumor (SNA + DLPS + clinical)	0.764	0.828	0.735	0.947	0.800
	Stroma (SNA + DLPS + clinical)	**0.907**	0.892	0.889	0.895	0.792
	iTILs (DLPS + clinical)	0.769	0.805	0.750	0.868	0.700
	sTILs (DLPS + clinical)	0.838	0.840	0.773	0.919	0.778
	TILs (SNA + DLPS + clinical)	0.803	0.857	0.783	0.947	0.812
Clinical	ER	0.658	0.765	0.721	0.816	0.632
	ER%	0.668	0.776	0.702	0.868	0.667
	PR	0.669	0.795	0.700	0.921	0.750
	PR%	0.645	0.686	0.750	0.632	0.533
	CEP17	0.573	0.763	0.627	0.974	0.666
	HER2/CEP17 ratio	0.674	0.795	0.700	0.921	0.750
Tiles count	Tumor	0.615	0.693	0.703	0.684	0.520
	Stroma	0.680	0.787	0.661	0.974	0.833
	iTILs	0.651	0.688	0.846	0.579	0.556
	sTILs	0.651	0.623	0.826	0.500	0.513
	TILs	0.596	0.671	0.627	0.721	0.666
Relative	TSR	0.576	0.769	0.660	0.921	0.667
	iLTR	0.562	0.770	0.842	0.710	0.400
	sTILR	0.571	0.758	0.632	0.947	0.600
	TISR	0.587	0.743	0.625	0.917	0.625
	LD	0.518	0.761	0.648	0.921	0.625
Latest work	IMPRESS (H&E only) [31]	0.812	0.827	0.906	0.761	0.698
	Pathologists’ features [31]	0.788	0.782	0.870	0.711	0.645
	LTR [63]	0.544	0.758	0.632	0.947	0.600

HER2, human epidermal growth factor receptor 2; AUC, area under the receiver operating characteristic curve; PPV, positive predictive value; NPV, negative predictive value; SNA, social network analysis; DLPS, deep-learning-derived pathologic complete response score; iTILs, intratumoral tumor-infiltrating lymphocytes; sTILs, stromal tumor-infiltrating lymphocytes; TILs, tumor-infiltrating lymphocytes; ER, estrogen receptor; PR, progesterone receptor; CEP17, centromere of chromosome 17; TSR, tumor-to-stroma ratio; iLTR, intratumoral tumor-infiltrating lymphocytes-to-tumor ratio; sTILR, stromal tumor-infiltrating lymphocyte ratio; TISR, tumor-infiltrating lymphocytes-to-stroma ratio; LD, lymphocyte density; LTR, lymphocyte-to-tumor ratio; H&E, hematoxylin and eosin; IMPRESS, IMage-based Pathological REgistration and Segmentation Statistics

Published approaches were also included for benchmarking, such as the IMPRESS method and pathologist-defined features reported by Huang et al., along with the more recent lymphocyte-to-tumor ratio model proposed by Aswolinskiy et al. Our proposed models and the baselines implemented in this study were evaluated using the same WSIs and outcome labels for direct comparison. For previously published methods, we report the performance metrics as described in the original studies on the IMPRESS HER2+ dataset; because we did not reimplement these methods, cross-study comparisons should be interpreted with caution. Our results indicate that tissue-specific models based on our framework achieve consistently strong performance across all evaluated baselines in the 5 tissue compartments. The model derived from the stromal compartment achieved the highest overall performance, with an AUC of 0.907, an F1 score of 0.892, and a recall of 0.895. To further assess the statistical significance of performance differences, pairwise comparisons of AUCs were conducted using the DeLong test, with corresponding 95% confidence intervals reported in Section S7 of Data File S1.

Compared with handcrafted tile-count features and simple compartmental ratios, our tissue-specific models consistently achieved higher AUCs while maintaining a more balanced precision–recall trade-off. Notably, the proposed framework outperformed pathologist-defined features in 4 of the 5 tissue compartments and demonstrated favorable performance relative to the IMPRESS model across most evaluation metrics, with the largest performance gains observed in the stromal compartment. Importantly, all tissue-specific models were developed using routinely available H&E-stained slides without reliance on specialized IHC staining or molecular profiling. This suggests the robustness and potential scalability of the proposed framework.

### Univariate analysis

To explore the independent predictive ability of extracted features, univariate statistical analyses were performed on both SNA-derived graph features and DLPSs, separately in the Yale Response and IMPRESS HER2+ datasets. Differences between the pCR and non-pCR groups were assessed using the Mann–Whitney *U* test, with statistical significance determined based on BH-FDR-adjusted *P* values.

**Fig. 5.**
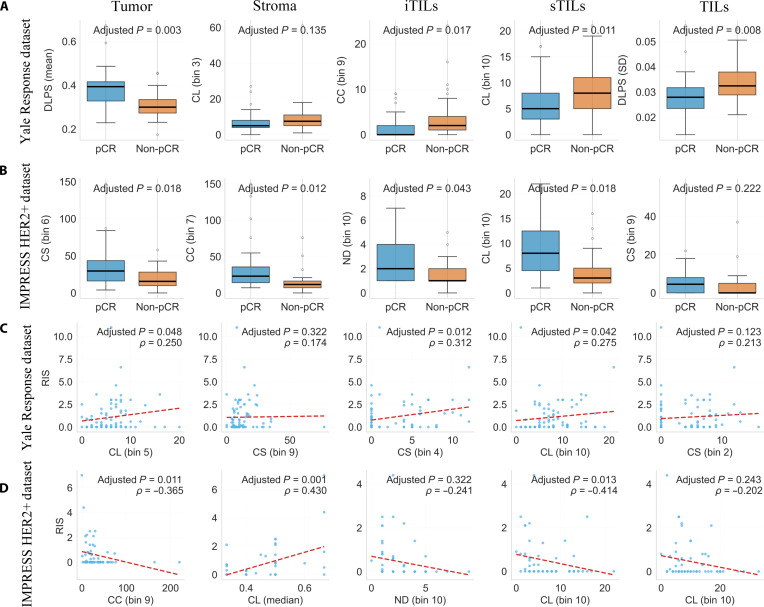
Univariate statistical analyses across 5 tissue compartments (tumor, stroma, intratumoral tumor-infiltrating lymphocytes [iTILs], stromal tumor-infiltrating lymphocytes [sTILs], and tumor-infiltrating lymphocytes [TILs]) in the Yale Response and IMPRESS HER2+ datasets. (A and B) Boxplots comparing selected features between the pathologic complete response (pCR) and non-pCR groups. *P* values were adjusted for multiple testing using the Benjamini–Hochberg false discovery rate (BH-FDR) procedure; FDR-adjusted *P* values are displayed. (C and D) Scatterplots showing associations between selected features and RIS. Red dashed lines indicate fitted lines for visualization. *ρ* denotes Spearman’s rank correlation coefficient. ND, node degree, CC, closeness centrality; CL, clustering coefficient; CS, community structure; RIS, residual infiltration size; IMPRESS, IMage-based Pathological REgistration and Segmentation Statistics.

Representative results across the 5 tissue compartments are shown in Fig. 5A and B, and the complete statistical results are provided in Data File S3. Several SNA features exhibited statistically significant differences between response groups. In the iTIL compartment, the frequency in bin 9 of the CC distribution was higher in the non-pCR group than in the pCR group (adjusted *P* = 0.017). DLPS features from the tumor and TIL compartments also showed significant differences between response groups in the Yale Response dataset. In parallel, Spearman’s rank correlation analysis was conducted to assess associations between individual features and RIS. Representative correlations are presented in Fig. 5C and D, with detailed results summarized in Data File S4. Notably, in the IMPRESS HER2+ dataset, the median CL in the stromal compartment showed a positive correlation with RIS (*ρ* = 0.430, adjusted *P* = 0.001), whereas several other features demonstrated weaker or nonsignificant associations. Together, these results indicate that both DLPS and SNA-derived graph features provide complementary information. While DLPS summarizes semantic patterns learned from tissue tiles, graph-based features quantify spatial organization that may be associated with immune infiltration patterns and TME structure.

### Feature attribution via LASSO

**Fig. 6.**
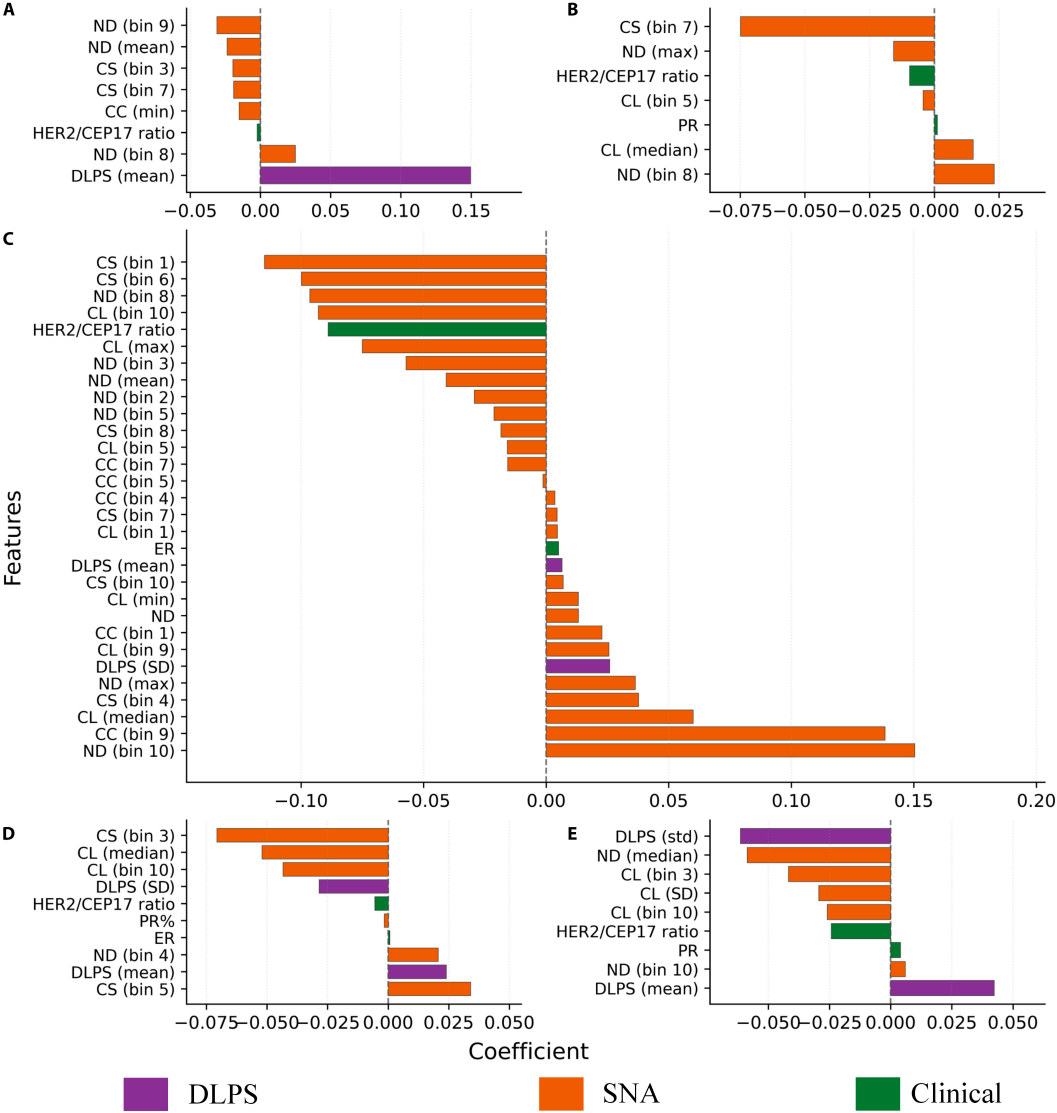
Tissue-specific visualization of least absolute shrinkage and selection operator (LASSO)-selected feature coefficients across 5 tissue compartments. Subplots show selected features in (A) tumor, (B) stroma, (C) intratumoral tumor-infiltrating lymphocytes (iTILs), (D) stromal tumor-infiltrating lymphocytes (sTILs), and (E) tumor-infiltrating lymphocytes (TILs) compartments. Features are grouped by source (social network analysis [SNA], deep-learning-derived pathologic complete response score [DLPS], and clinical variables), with bar lengths representing standardized LASSO weights.

To enhance the interpretability of our tissue-specific predictive models, we examined the features selected by the LASSO algorithm across the 5 tissue compartments. The selected features and their corresponding coefficients are shown in Fig. 6. Each model was trained using a set of 99 input features, including 91 graph-based structural descriptors derived from SNA, 2 semantic scores produced by the DL pipeline, and 6 clinical variables. Given the distinct biological roles of tumor, stromal, and immune compartments, we anticipated that the relative importance of semantic, structural, and clinical features would differ across compartments rather than remain uniform.

In the tumor compartment, the mean DLPS value emerged as one of the most influential retained features, indicating that semantic patterns in tumor tiles carry substantial prognostic value. ND and CC were also selected, characterizing the spatial compactness and connectivity of tumor regions, as reported in prior studies [53]. CS-related features further suggested that tumor subregion organization holds relevance for treatment response. The HER2/CEP17 ratio was the only clinical variable retained, complementing image-derived features.

In the stromal compartment, graph-derived features contributed prominently to the model. ND and CC were again selected with substantial weights, capturing the spatial configuration of stromal tissue. These structural patterns have been associated with immune-excluded microenvironmental states and treatment response in prior studies [54,55]. The standard deviation of DLPS was also retained, highlighting the predictive relevance of semantic heterogeneity in stromal compartments. Although clinical variables such as ER and PR contributed less prominently, their inclusion suggests potential complementarity when combined with structural and semantic information.

In the iTIL compartment, most selected features were graph-based. ND and CC ranked among the top contributors, suggesting that the density and clustering of iTILs may be associated with therapeutic responsiveness [56]. Conversely, one of the CS histogram bins exhibited a negative coefficient, potentially reflecting a less organized immune spatial architecture, which has been linked to distinct tumor-immune microenvironmental states in prior spatial profiling studies [57]. Clinical variables including PR status and HER2/CEP17 ratio were also selected, although their contributions were relatively limited.

In the sTIL compartment, the most important features included a CS histogram count and the mean value of DLPS. sTILs are established biomarkers in breast cancer and are routinely evaluated in stromal regions [58]. CC and the variability of DLPS were also retained, indicating that both spatial cohesion and semantic heterogeneity are relevant in this context. Clinical variables such as ER status and HER2/CEP17 ratio were included with moderate importance.

In the TIL compartment, the mean value of DLPS remained the dominant predictor. This result suggests that high-level semantic patterns in lymphocyte-rich compartments may be associated with treatment response. The standard deviation of DLPS was also retained, with a negative coefficient, suggesting that lower semantic variability may be associated with more homogeneous tissue patterns; mechanistic interpretation requires further validation. Graph-based metrics such as ND and CL were additionally selected, highlighting the role of immune cell aggregation in modulating therapeutic response. Clinical features were included but contributed minimally.

Overall, the selected features reflect distinct compartment-specific contributions: the tumor model combined strong semantic signals (DLPS) with selected structural descriptors, whereas the stromal model was predominantly characterized by graph-derived structural features, and immune-related compartments captured both semantic and spatial organizational patterns. This pattern is qualitatively consistent with representative tissue graphs (Fig. 4) and prior spatial TME studies [59]. The integration of structural metrics, semantic representations, and clinical variables facilitated interpretable, tissue-specific modeling of NAC response in HER2+BC. These findings suggest compartment-specific associations between selected features and response.

### Impact of training data size on generalization performance

To further assess the generalization capability of our method, we conducted a sample ablation experiment using the complete set of 3 feature types, which included DLPS, SNA-derived features, and clinical variables. Tissue-specific models were trained using progressively larger portions of the training data, namely, 20%, 40%, 60%, and 80%, and evaluated on the same external validation dataset employed in the main experiment. The modeling pipeline and evaluation procedures were identical to those described previously and are not repeated here.

**Fig. 7.**
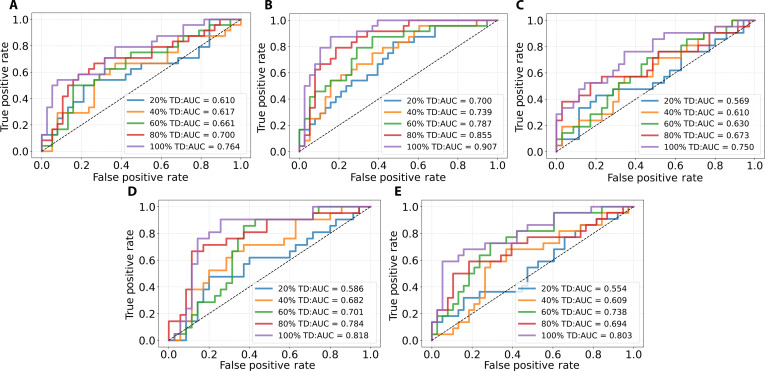
Receiver operating characteristic (ROC) curves of tissue-specific models trained on different proportions of the training data and evaluated on the external IMPRESS human epidermal growth factor receptor 2-positive (HER2+) dataset. (A) to (E) correspond to the tumor, stroma, intratumoral tumor-infiltrating lymphocytes (iTILs), stromal tumor-infiltrating lymphocytes (sTILs), and tumor-infiltrating lymphocytes (TILs), respectively. Each plot shows model performance under 20%, 40%, 60%, and 80% training data. TD, training data; IMPRESS, IMage-based Pathological REgistration and Segmentation Statistics.

As presented in Fig. 7, model performance improved consistently with increased training data across all tissue compartments. Notably, even when trained with only 20% of the available data, the models exhibited meaningful predictive capability. In the stromal compartment, for instance, the model achieved an AUC of 0.700, demonstrating stable performance under limited supervision. Across all training proportions, the stromal compartment consistently yielded the highest predictive scores. Complete quantitative results, including F1 score, positive predictive value, recall, and negative predictive value across all training levels and tissue compartments, are provided in Section S8 of Data File S1. These results demonstrate the robustness and data efficiency of the proposed framework, suggesting that it could be useful for pCR prediction, particularly in low-data scenarios.

## Discussion

While IHC staining remains a routine tool for assessing response to NAC, its reliance on additional laboratory resources, cost, and tissue availability limits scalability in real-world settings. This has accelerated interest in DL methods based on standard H&E slides; however, many existing approaches primarily operate on independent tiles and therefore underuse spatial context, constraining biological interpretability and model robustness. Although graph-based models have been proposed at the cellular or patch level, relatively few methods explicitly capture mesoscale organization through biologically defined tissue compartments. Importantly, our framework deliberately operates at the tissue level to capture mesoscale spatial organization that is robust, interpretable, and compatible with routine H&E-stained slides, rather than explicitly modeling fine-grained single-cell interactions, which typically require complex cell segmentation pipelines.

To address these limitations, we developed a tissue-specific modeling framework that integrates spatial features from tissue graphs, semantic representations from DL, and standard clinical variables. The analysis focused on 5 biologically defined tissue compartments, including tumor, stroma, iTILs, sTILs, and their union (TILs), which were delineated through tissue classification and spatial localization. For each compartment, an independent graph was constructed and SNA was applied to extract interpretable features characterizing local spatial structure. In parallel, semantic features were obtained using a CLAM-based encoder tailored to each compartment, and all features were integrated into a unified hierarchical input for predictive modeling.

External validation on the IMPRESS HER2+ dataset demonstrated the strong predictive performance of the proposed framework. The stromal compartment achieved the highest AUC of 0.907, and all compartments exceeded 0.750, suggesting the potential generalizability of the approach. Notably, the stromal and sTIL compartments outperformed the evaluated baselines by 9.5% and 2.6%, respectively, suggesting the potential added value of integrating spatial context beyond conventional tumor-centric representations. Robustness was further supported by sample ablation experiments. Even with only 20% of the training data, the stromal model maintained an AUC of 0.700, suggesting that stromal spatial characteristics provide strong predictive signals. To assess the robustness of the modular pipeline, we evaluated the framework on an independent external cohort and conducted sample ablation analyses, which collectively suggest the robustness of the proposed workflow. These results suggest that stromal architecture contains information that may be associated with treatment response in HER2+BC.

To enhance interpretability, we conducted univariate analyses and correlation studies with RIS. Univariate and correlation analyses revealed compartment-specific associations of selected SNA metrics (e.g., CS/CC/CL/ND-related bins) and DLPS with pCR and RIS, rather than uniform effects across all tissue compartments. Features such as selected SNA metrics demonstrated marked differences or consistent trends in specific compartments, suggesting that mesoscale spatial organization may be associated with treatment sensitivity. LASSO-based feature selection further supported these findings. In the tumor and stromal compartments, a subset of SNA-derived metrics (including ND and CC) was more frequently retained, suggesting that higher tissue connectivity and spatial centralization may be negatively correlated with pCR. In immune-related compartments, including iTILs and TILs, CC- and CS-related features were frequently selected, which may serve as proxies for local immune aggregation and spatial organization. These biological interpretations are speculative and are provided to aid histopathologic understanding; they require dedicated biological validation in future studies. Overall, DL-derived features contributed prominently in the tumor compartment, whereas graph-based SNA metrics were frequently retained in immune-related compartments, underscoring the tissue-specific relevance of semantic and structural representations.

Recent spatial modeling approaches in computational pathology have also explored graph neural networks, cell-level graph models, and transformer-based whole-slide learning frameworks, which can capture complex spatial dependencies but often require fine-grained cell segmentation, large annotated datasets, or high-capacity black-box models [60–62]. In contrast, our framework deliberately operates at the tissue-compartment and mesoscale level, prioritizing interpretability, robustness, and clinical feasibility over architectural complexity. Within the current landscape of spatial computational pathology, our approach complements cell-level and end-to-end DL frameworks by providing an interpretable and workflow-compatible alternative for modeling mesoscale tissue organization from routine H&E-stained slides. Importantly, these findings are preliminary and do not constitute direct mechanistic validation of immune activity or drug response. Clinical variables such as ER status were selected in some models but showed limited consistency. Incorporating clinical variables together with imaging-derived features may introduce redundancy or multicollinearity; therefore, LASSO-based feature selection was applied within each tissue-specific model to retain only variables providing additional predictive value beyond SNA/DLPS features. These findings suggest that no single feature type dominates across tissue compartments; instead, structural, semantic, and clinical features provide complementary, tissue-dependent contributions to treatment response prediction, supporting the rationale of tissue-specific modeling in HER2+BC.

Despite these promising results, several limitations remain. First, graph construction and SNA metrics were based on tissue-level classification rather than single-cell resolution, which improves interpretability and efficiency but may introduce bias and does not explicitly model finer spatial interactions such as direct lymphocyte–tumor contact. Future studies could incorporate cell segmentation and classification to enhance biological fidelity. Second, the current study focused solely on HER2+BC. Although the framework is conceptually generalizable, its applicability to other breast cancer subtypes and tumor types requires further validation. Third, the overall sample size was relatively limited, which may affect model stability and increase the risk of cohort-specific associations, although external validation was performed. Larger, multi-institutional datasets are needed to confirm the scalability, generalizability, and clinical relevance of the proposed approach in real-world settings. In addition, formal cross-institutional heterogeneity analysis and statistical power calculations were not performed, as the study focused on predictive modeling rather than hypothesis-driven inference. Instead, robustness was evaluated through independent external validation and sample ablation analyses.

The proposed framework was developed and validated using digitized WSIs from 2 available public datasets, and its performance may be affected by variations in scanner types, staining protocols, and other preanalytical factors across institutions. Such domain shifts remain a general challenge for computational pathology models and warrant further investigation. In addition, while the modular pipeline improves interpretability and flexibility, further validation of clinical utility, including prospective multicenter studies and evaluation under domain shift, is necessary before any clinical application can be considered.

## Conclusion

This study introduces a tissue-specific predictive framework that integrates structural, semantic, and clinical features to estimate pCR to NAC in HER2+BC. By modeling intratissue organization across distinct compartments of the TME, the framework enhances biological interpretability and highlights spatial patterns associated with treatment sensitivity that are often missed by conventional DL-based approaches. Independent validation confirmed its consistent and robust predictive performance, with the stromal compartment achieving the highest predictive performance. Notably, the method relies entirely on routinely collected H&E-stained WSIs, requiring no specialized staining or manual annotation, and maintains strong generalizability even when training data are limited. Feature attribution analysis revealed that graph-based metrics, particularly CL and ND, were consistently associated with treatment response across stromal and immune-related compartments. These features may serve as proxies for spatial connectivity and immune cell aggregation, motivating future mechanistic and experimental validation of the structural patterns captured by the framework. Overall, tissue-specific modeling based on routine H&E slides demonstrates potential for pCR estimation, yet additional validation across multiple institutions is necessary prior to clinical use.

## Data Availability

The Yale Response dataset is available at The Cancer Imaging Archive (TCIA) (DOI: 10.7937/E65C-AM96). The IMPRESS HER2+ dataset is available at https://tinyurl.com/IMPRESS-DATA, as described in the original publication.

## Supplementary Materials

Supplementary 1 Sections S1 to S8 Figs. S1 and S2 Tables S1 to S7 Data Files S1 to S5 References [64–69]
