# Supplementary material for: Hierarchical Tissue-Specific Modeling of Pathology Images Predicts Response in HER2+ Breast Cancer
Source: Cyborg Bionic Syst. 2026 Apr 22;7:0554. doi: 10.34133/cbsystems.0554 (PMC13100347; doi:10.34133/cbsystems.0554)
Supplement: Supplementary 1 — Sections S1 to S8 Figs. S1 and S2 Tables S1 to S7 Data Files S1 to S5 References [64–69] [file cbsystems.0554.f1.zip › Data files S1_.docx]

**Supplementary Section S1. CNN-based tissue classification**

**S1.1 Tumor classification**

We analyzed 195 whole slide images (WSIs) at 40× magnification from The Cancer Genome Atlas, using corresponding annotation masks provided by Janowczyk et al. [66]. These annotation masks, derived from expert pathologist labels, delineated tumor regions at the pixel level and were aligned with the WSIs to assign each tile a label of either tumor or stroma. WSIs were segmented into tile images with dimensions of 350×350×3 pixels. Otsu’s thresholding was applied to exclude tiles with less than 26 percent tissue content. Tumor regions were annotated based on the mask images that delineated tumor areas. Stroma was defined as non-fat tissue located outside these annotated tumor regions within pathology images, excluding non-informative areas eliminated during preprocessing. Spatial coordinates from the annotation masks were aligned with the WSIs to assign each tile a label of either tumor or stroma. This process resulted in 2,260,684 labeled tiles. The 195 patients were split into training, validation, and test sets. The training set consisted of 157 patients, accounting for 1,708,303 tiles; the validation set included 18 patients with 201,135 tiles; and the test set contained 20 patients with 303,019 tiles. To alleviate computational demands, we randomly selected 20% of the tiles from each patient in the training set, yielding a total of 351,246 tiles. Tumor versus stroma classification was conducted using a modified Xception model adapted via transfer learning [67]. The final fully connected layer was replaced by a global max pooling layer and two output neurons with SoftMax activation, enhancing input flexibility, reducing model parameters, and improving generalization. The model was trained using stochastic gradient descent with a learning rate of 0.01, a momentum of 0.9, a batch size of 128, and the cross-entropy loss function.

**S1.2 Necrosis classification**

To reduce false positives arising from morphological similarities between necrotic cell nuclei and intratumoral tumor-infiltrating lymphocytes, we developed a convolutional neural network (CNN) for necrosis identification. The dataset comprised 2,237 image tiles (500×500×3 pixels) from LUAD cases at 20× magnification, sourced from a publicly available database [56]. To enhance robustness and generalizability, we employed a sliding window approach with 100×100 pixel windows and a stride of 100 pixels. This process yielded a total of 55,925 tiles. A tile was labeled as necrotic if more than 20 percent of its pixels contained necrotic tissue [68]. A fine-tuned VGG-16 architecture was employed for transfer learning, and model evaluation was conducted using stratified five-fold cross-validation [69]. Each fold contained 44,740 training tiles and 11,185 testing tiles, corresponding to balanced distributions of patients and biopsy cassettes across folds. The network was optimized by substituting the fully connected layers with a global max pooling layer, a dropout layer with a rate of 0.5, a dense layer of 128 neurons activated by ReLU, and a SoftMax output layer. Training was performed using the Adam optimizer with a learning rate of 0.0001, a batch size of 16, and 100 training epochs.

**S1.3 Lymphocyte classification**

To analyze the tumor immune microenvironment, we implemented a deep learning model for lymphocyte identification. This model was trained and validated using public datasets of LUAD (10 patients) and BRCA (18 patients) at 20× magnification, totaling 34,404 tiles, each with a resolution of 500×500×3 pixels. Lymphocyte infiltration status was determined based on the central 100×100 pixel region of each tile. Tiles were labeled as infiltrated if lymphocytes were visibly present within the central 100×100 pixel region, based on expert annotation. Stratified five-fold cross-validation was conducted, with each fold consisting of 27,523 training tiles and 6,881 testing tiles. The model employed the same architecture and hyperparameters as used for necrosis detection (fine-tuned VGG-16, Adam optimizer, learning rate = 0.0001, batch size = 16, 100 epochs)

**S1.4 Tissue compartment** **identification results**

During the generation of tissue type annotation images (TTA-images), we developed and trained three separate tissue classification models, each dedicated to a specific task.The modeling procedure for each task is described as follows.

For tumor classification, the Xception architecture was subjected to full-layer fine-tuning with an early stopping strategy to prevent overfitting. Training converged at epoch 25. Evaluation on the validation and test sets yielded classification accuracies of 0.865 and 0.860, respectively, with corresponding area under the curve (AUC) values of 0.927 and 0.917. The stable performance across datasets demonstrates strong generalizability and validates the sampling approach. To address magnification inconsistencies between the Yale Response and IMPRESS HER2-positive datasets (20×) and the model’s requirement for 40× input resolution, neoadjuvant chemotherapy (NAC) images were upsampled from 175×175×3 to 350×350×3 using bilinear interpolation.

The VGG-16 model was employed for necrosis classification and was similarly fine-tuned. Stratified five-fold cross-validation produced a mean classification accuracy of 0.964 ± 0.035. Final predictions were generated through ensemble averaging of probabilities across the five validation folds. The same modeling framework was applied for lymphocyte classification, resulting in a mean accuracy of 0.914 ± 0.031. Final outputs were again derived by averaging the predictions from the cross-validated models.

**Supplementary Section S2. Sensitivity analysis of graph construction parameters based on SNA features**

To assess the robustness of graph construction parameters, we evaluated nine combinations of the affinity propagation preference parameter, which controls clustering granularity (P = −100, −50, −20), and the K-nearest neighbor parameter, which defines local graph connectivity (K = 4, 5, 6), using SNA-derived features only. Each tissue compartment was modeled independently, and performance was assessed in terms of AUC, F1 score, positive predictive value (PPV), recall, and negative predictive value (NPV).

Table S1. Performance metrics across graph construction parameters and tissue compartments under 5-fold stratified cross-validation on the training set of the Yale Response Dataset.

| Compartments | K | P | AUC | F1 score | PPV | Recall | NPV |
| --- | --- | --- | --- | --- | --- | --- | --- |
| Tumor | 4 | -20 | 0.727 ± 0.098 | 0.707 ± 0.103 | 0.690 ± 0.093 | 0.779 ± 0.249 | 0.773 ± 0.082 |
|  |  | -50 | **0.819 ± 0.101** | 0.762 ± 0.136 | 0.708 ± 0.099 | 0.839 ± 0.192 | 0.805 ± 0.105 |
|  |  | -100 | 0.730 ± 0.085 | 0.664 ± 0.087 | 0.608 ± 0.086 | 0.753 ± 0.162 | 0.731 ± 0.083 |
|  | 5 | -20 | 0.711 ± 0.116 | 0.644 ± 0.083 | 0.612 ± 0.103 | 0.721 ± 0.177 | 0.707 ± 0.072 |
|  |  | -50 | 0.693 ± 0.083 | 0.646 ± 0.055 | 0.576 ± 0.031 | 0.746 ± 0.134 | 0.711 ± 0.031 |
|  |  | -100 | 0.770 ± 0.130 | 0.690 ± 0.119 | 0.715 ± 0.061 | 0.696 ± 0.205 | 0.745 ± 0.100 |
|  | 6 | -20 | 0.695 ± 0.071 | 0.529 ± 0.154 | 0.603 ± 0.136 | 0.550 ± 0.280 | 0.672 ± 0.091 |
|  |  | -50 | 0.693 ± 0.047 | 0.624 ± 0.077 | 0.599 ± 0.094 | 0.693 ± 0.187 | 0.700 ± 0.066 |
|  |  | -100 | 0.806 ± 0.087 | **0.785 ± 0.086** | **0.712 ± 0.113** | **0.889 ± 0.108** | **0.813 ± 0.074** |
| Stroma | 4 | -20 | 0.759 ± 0.124 | 0.678 ± 0.138 | 0.629 ± 0.088 | 0.800 ± 0.228 | 0.753 ± 0.107 |
|  |  | -50 | **0.776 ± 0.111** | 0.682 ± 0.111 | 0.665 ± 0.082 | 0.734 ± 0.229 | 0.747 ± 0.078 |
|  |  | -100 | 0.750 ± 0.142 | 0.659 ± 0.155 | **0.671 ± 0.054** | 0.724 ± 0.266 | 0.725 ± 0.085 |
|  | 5 | -20 | 0.710 ± 0.053 | 0.635 ± 0.032 | 0.664 ± 0.100 | 0.643 ± 0.143 | 0.697 ± 0.020 |
|  |  | -50 | 0.732 ± 0.096 | **0.716 ± 0.105** | 0.591 ± 0.100 | 0.786 ± 0.186 | 0.753 ± 0.081 |
|  |  | -100 | 0.685 ± 0.074 | 0.701 ± 0.062 | 0.586 ± 0.073 | **0.914 ± 0.193** | **0.798 ± 0.070** |
|  | 6 | -20 | 0.740 ± 0.091 | 0.609 ± 0.118 | 0.629 ± 0.066 | 0.657 ± 0.259 | 0.713 ± 0.104 |
|  |  | -50 | 0.751 ± 0.086 | 0.673 ± 0.081 | 0.646 ± 0.078 | 0.705 ± 0.121 | 0.724 ± 0.054 |
|  |  | -100 | 0.733 ± 0.088 | 0.631 ± 0.126 | 0.631 ± 0.132 | 0.695 ± 0.257 | 0.714 ± 0.077 |
| iTILs | 4 | -20 | 0.750 ± 0.054 | 0.682 ± 0.038 | 0.630 ± 0.058 | 0.783 ± 0.168 | 0.740 ± 0.073 |
|  |  | -50 | **0.805 ± 0.096** | **0.721 ± 0.074** | **0.689 ± 0.054** | 0.767 ± 0.114 | **0.752 ± 0.065** |
|  |  | -100 | 0.787 ± 0.128 | 0.698 ± 0.082 | 0.652 ± 0.057 | 0.767 ± 0.146 | 0.749 ± 0.084 |
|  | 5 | -20 | 0.710 ± 0.086 | 0.679 ± 0.058 | 0.600 ± 0.061 | **0.858 ± 0.132** | 0.741 ± 0.072 |
|  |  | -50 | 0.748 ± 0.072 | 0.645 ± 0.050 | 0.654 ± 0.063 | 0.652 ± 0.113 | 0.701 ± 0.053 |
|  |  | -100 | 0.733 ± 0.057 | 0.682 ± 0.072 | 0.618 ± 0.039 | 0.767 ± 0.125 | 0.733 ± 0.060 |
|  | 6 | -20 | 0.767 ± 0.108 | 0.655 ± 0.105 | 0.606 ± 0.066 | 0.729 ± 0.192 | 0.725 ± 0.099 |
|  |  | -50 | 0.774 ± 0.066 | 0.690 ± 0.113 | 0.691 ± 0.108 | 0.729 ± 0.152 | 0.747 ± 0.062 |
|  |  | -100 | 0.730 ± 0.054 | 0.655 ± 0.070 | 0.570 ± 0.065 | 0.790 ± 0.191 | 0.723 ± 0.073 |
| sTILs | 4 | -20 | 0.710 ± 0.053 | 0.635 ± 0.032 | 0.664 ± 0.100 | 0.643 ± 0.143 | 0.697 ± 0.020 |
|  |  | -50 | 0.733 ± 0.088 | 0.631 ± 0.126 | 0.631 ± 0.132 | 0.695 ± 0.257 | 0.714 ± 0.077 |
|  |  | -100 | 0.750 ± 0.142 | 0.659 ± 0.155 | 0.671 ± 0.054 | 0.724 ± 0.266 | 0.725 ± 0.085 |
|  | 5 | -20 | **0.759 ± 0.124** | 0.678 ± 0.138 | 0.629 ± 0.088 | 0.800 ± 0.228 | 0.753 ± 0.107 |
|  |  | -50 | 0.732 ± 0.096 | **0.716 ± 0.105** | 0.591 ± 0.100 | 0.786 ± 0.186 | 0.753 ± 0.081 |
|  |  | -100 | 0.685 ± 0.074 | 0.701 ± 0.062 | 0.586 ± 0.073 | **0.914 ± 0.193** | **0.798 ± 0.070** |
|  | 6 | -20 | 0.740 ± 0.091 | 0.609 ± 0.118 | 0.629 ± 0.066 | 0.657 ± 0.259 | 0.713 ± 0.104 |
|  |  | -50 | 0.751 ± 0.086 | 0.673 ± 0.081 | 0.646 ± 0.078 | 0.705 ± 0.121 | 0.724 ± 0.054 |
|  |  | -100 | 0.776 ± 0.111 | 0.682 ± 0.111 | **0.665 ± 0.082** | 0.734 ± 0.229 | 0.747 ± 0.078 |
| TILs | 4 | -20 | 0.711 ± 0.074 | 0.671 ± 0.113 | 0.610 ± 0.081 | 0.775 ± 0.217 | 0.744 ± 0.074 |
|  |  | -50 | **0.812 ± 0.065** | **0.734 ± 0.086** | **0.685 ± 0.100** | **0.827 ± 0.128** | 0.771 ± 0.064 |
|  |  | -100 | 0.691 ± 0.102 | 0.655 ± 0.056 | 0.597 ± 0.065 | 0.778 ± 0.174 | 0.723 ± 0.064 |
|  | 5 | -20 | 0.718 ± 0.073 | 0.678 ± 0.050 | 0.633 ± 0.036 | 0.759 ± 0.157 | 0.735 ± 0.063 |
|  |  | -50 | 0.750 ± 0.095 | 0.683 ± 0.086 | 0.631 ± 0.074 | 0.728 ± 0.139 | 0.728 ± 0.080 |
|  |  | -100 | 0.756 ± 0.095 | 0.691 ± 0.122 | 0.656 ± 0.064 | 0.789 ± 0.262 | **0.772 ± 0.099** |
|  | 6 | -20 | 0.731 ± 0.052 | 0.674 ± 0.073 | 0.629 ± 0.083 | 0.775 ± 0.188 | 0.735 ± 0.057 |
|  |  | -50 | 0.733 ± 0.066 | 0.689 ± 0.062 | 0.657 ± 0.085 | 0.754 ± 0.162 | 0.742 ± 0.078 |
|  |  | -100 | 0.737 ± 0.119 | 0.662 ± 0.126 | 0.617 ± 0.077 | 0.823 ± 0.210 | 0.748 ± 0.089 |

Note: The best result in each column is highlighted in **bold**. P: Preference.

Table S2. Performance metrics across graph construction parameters and tissue compartments on the IMPRESS HER2+ external validation dataset.

| Compartments | K | Preference | AUC | F1 score | PPV | Recall | NPV |
| --- | --- | --- | --- | --- | --- | --- | --- |
| Tumor | 4 | -20 | 0.658 | 0.776 | 0.702 | 0.868 | 0.647 |
|  |  | -50 | 0.654 | 0.750 | 0.621 | 0.947 | 0.500 |
|  |  | -100 | 0.657 | 0.760 | 0.613 | **1.000** | 0.500 |
|  | 5 | -20 | 0.627 | **0.768** | 0.623 | **1.000** | **0.667** |
|  |  | -50 | 0.668 | 0.747 | 0.689 | 0.816 | 0.579 |
|  |  | -100 | **0.684** | 0.759 | 0.732 | 0.789 | 0.609 |
|  | 6 | -20 | 0.623 | 0.747 | 0.689 | 0.816 | 0.579 |
|  |  | -50 | 0.664 | 0.720 | 0.730 | 0.711 | 0.556 |
|  |  | -100 | 0.660 | 0.704 | **0.758** | 0.658 | 0.548 |
| Stroma | 4 | -20 | 0.668 | **0.826** | 0.704 | **1.000** | **0.900** |
|  |  | -50 | **0.793** | 0.795 | 0.700 | 0.921 | 0.714 |
|  |  | -100 | 0.765 | 0.789 | 0.789 | 0.789 | 0.654 |
|  | 5 | -20 | 0.615 | 0.756 | 0.654 | 0.895 | 0.583 |
|  |  | -50 | 0.663 | 0.687 | **0.793** | 0.605 | 0.543 |
|  |  | -100 | 0.663 | 0.739 | 0.630 | 0.895 | 0.500 |
|  | 6 | -20 | 0.662 | 0.737 | 0.737 | 0.737 | 0.577 |
|  |  | -50 | 0.633 | 0.729 | 0.660 | 0.816 | 0.529 |
|  |  | -100 | 0.576 | 0.700 | 0.667 | 0.737 | 0.500 |
| iTILs | 4 | -20 | 0.542 | 0.618 | 0.700 | 0.553 | 0.455 |
|  |  | -50 | **0.649** | 0.759 | 0.732 | 0.789 | 0.591 |
|  |  | -100 | 0.615 | 0.744 | 0.653 | 0.865 | 0.538 |
|  | 5 | -20 | 0.624 | 0.696 | **0.750** | 0.649 | 0.533 |
|  |  | -50 | 0.596 | 0.753 | 0.667 | 0.865 | 0.571 |
|  |  | -100 | 0.619 | 0.709 | 0.651 | 0.778 | 0.500 |
|  | 6 | -20 | 0.580 | 0.758 | 0.632 | **0.947** | 0.500 |
|  |  | -50 | 0.580 | 0.742 | 0.647 | 0.868 | 0.500 |
|  |  | -100 | 0.613 | **0.773** | 0.667 | 0.919 | **0.636** |
| sTILs | 4 | -20 | 0.670 | 0.737 | 0.718 | 0.757 | 0.545 |
|  |  | -50 | 0.669 | 0.737 | 0.683 | 0.800 | 0.529 |
|  |  | -100 | 0.635 | 0.711 | 0.692 | 0.730 | 0.500 |
|  | 5 | -20 | 0.662 | 0.767 | 0.673 | 0.892 | 0.583 |
|  |  | -50 | 0.655 | 0.696 | **0.750** | 0.649 | 0.517 |
|  |  | -100 | 0.662 | **0.774** | 0.643 | **0.973** | **0.600** |
|  | 6 | -20 | **0.684** | 0.771 | 0.696 | 0.865 | 0.500 |
|  |  | -50 | 0.612 | 0.727 | 0.627 | 0.865 | 0.400 |
|  |  | -100 | 0.607 | 0.734 | 0.690 | 0.784 | 0.526 |
| TILs | 4 | -20 | 0.601 | 0.742 | 0.647 | 0.868 | 0.500 |
|  |  | -50 | 0.667 | 0.768 | 0.623 | 1.000 | 0.500 |
|  |  | -100 | 0.571 | 0.699 | 0.644 | 0.763 | 0.444 |
|  | 5 | -20 | 0.643 | 0.677 | **0.786** | 0.595 | 0.529 |
|  |  | -50 | 0.676 | 0.771 | 0.696 | 0.865 | 0.625 |
|  |  | -100 | **0.683** | 0.776 | 0.688 | 0.892 | 0.643 |
|  | 6 | -20 | 0.591 | **0.779** | 0.649 | **0.974** | **0.667** |
|  |  | -50 | 0.566 | 0.763 | 0.627 | **0.974** | 0.500 |
|  |  | -100 | 0.603 | 0.714 | 0.652 | 0.789 | 0.471 |

Note: The best result in each column is highlighted in **bold**.


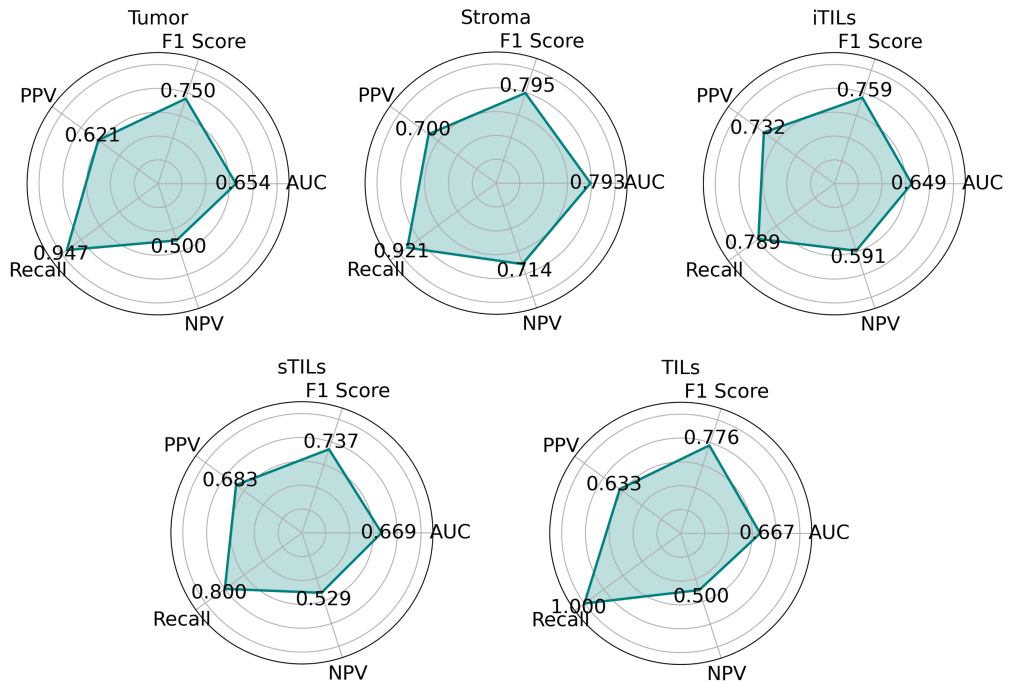


Fig. S1. Radar plots summarizing tissue-specific classification performance of spatial graphs under optimal hyperparameters (*K* = 4 neighbors, AP preference *P* = -50). Each plot corresponds to a distinct tissue compartment (tumor, stroma, iTILs, sTILs, and TILs) and reports five evaluation metrics: AUC, F1 score, PPV, recall, and negative predictive value (NPV).

**Supplementary Section S3. Formal definitions of SNA metrics**

**Node Degree (ND):** ND quantifies the number of direct connections each node has within the graph. In tissue graphs, a node with a high degree often represents a compartment that is spatially surrounded by multiple tissue clusters. In lymphocyte-rich areas such as TILs, this may reflect active immune infiltration or clustering of inflammatory cells.

**Clustering Coefficient (CL):** CL of a node *v* quantifies the likelihood that its neighbors are also connected.

$$\begin{aligned} {CL}_{v} = \frac{2T\left( v \right)}{\deg\left( v \right)\left( \deg\left( v \right)-1 \right)}\#\left( 1 \right) \end{aligned}$$

where T(***v***) is the number of triangles passing through node ***v*** and deg(***v***) is its degree. CL measures the extent to which a node’s neighbors are interconnected, thereby capturing the level of local cohesion in the network. Biologically, a high CL in lymphocyte compartments suggests that immune cells are not only densely populated but also form compact micro-clusters, which may reflect organized immune responses.

**Closeness Centrality (CC):** Measures how close a node is to all others in the graph based on shortest path distances.

$$\begin{aligned} CC\left( \boldsymbol{v} \right) = \frac{N-1}{\sum_{u=1}^{n-1} \mathrm{distance} \left( \boldsymbol{u},\boldsymbol{v} \right)} \#\left( 2 \right) \end{aligned}$$

where $\mathrm{distance}$(u, ***v***) is the shortest path length between nodes u and ***v***, and *N*-1 is the total number of nodes. Tissue compartments with high CC values are more centrally positioned within their compartment and may play a key structural or functional role, such as tumor cores that are actively interacting with surrounding tissues.

**Degree Centrality (DC):** Represents the normalized number of direct connections of a node.

$$\begin{aligned} DC\left( \boldsymbol{v} \right) = \frac{\deg\left( \boldsymbol{v} \right)}{N-1}\#\left( 3 \right) \end{aligned}$$

where deg(***v***) is the number of directly connected neighbors. DC normalizes the number of direct connections by the total number of possible connections, thereby highlighting nodes with extensive spatial interactions. In tumor graphs, a high DC value may indicate spatially dominant tumor nests, whereas in stromal compartments it may correspond to bridge-like structures that facilitate tumor–stroma communication.

**Katz Centrality (KC):** A measure of global influence that accounts for all paths in the network with exponential attenuation.

$$\begin{aligned} {KC(\boldsymbol{v}}_{\boldsymbol{i}}) = \alpha\sum_{v_{j\in N(\boldsymbol{v}_{\boldsymbol{i}})}} A_{ij}KC(\boldsymbol{v}_{\boldsymbol{j}})+\beta_{i}\#\left( 4 \right) \end{aligned}$$

where $A_{ij}$ is the adjacency matrix, *α* is the attenuation factor, and *β* is the bias term. KC incorporates both direct and indirect connections to recursively assess node influence. This metric enables the identification of compartments that are structurally or functionally significant, even if they are not directly connected to many others. For instance, a node with a moderate degree but linked to highly central compartments may play a mediating role in spatial organization or intercellular signaling.

**Network Density (NED):** The ratio of observed edges to all possible edges in the graph.

$$\begin{aligned} NED\left( G \right) = \frac{2M}{N\left( N-1 \right)}\#\left( 5 \right) \end{aligned}$$

where *M* is the number of observed edges and *N* is the number of nodes. NED, as a global graph metric, quantifies the ratio of observed to possible connections, thus reflecting the overall compactness of tissue organization. Higher density in iTILs or lymphocyte graphs may indicate concentrated immune localization, while lower density in stromal graphs may be indicative of loosely distributed tissue structures such as stroma or vasculature.

**Community Structure (CS):** CS identifies densely connected subgraphs within the network, revealing potential functional microenvironments within the slide. These communities may correspond to tumor nests, immune cell aggregates, or isolated stromal compartments adjacent to tumor boundaries.

**Supplementary Section S4. Network** **architecture and tile-level classification comparisons**

The Yale Response dataset was divided on a per-patient basis into a training set with 68 patients and an internal validation set with 17 patients. Three histopathology encoders, namely CLAM, UNI, and CONCH, were applied as feature extractors. CLAM and UNI produced 1024-dimensional tile-level embeddings, while CONCH generated 512-dimensional embeddings.

Based on the CNN-based tissue classification results described in Supplementary Section S1, all tiles from different sources were resized to 512×512 to standardize input resolution across magnifications. This procedure yielded 255,560 tiles for the training set and 61,996 tiles for the internal validation set. The IMPRESS HER2+ dataset was processed in the same manner and used exclusively for external evaluation, resulting in 410,492 tiles in total. No data from the external cohort were used during model training or hyperparameter tuning.

A multi-layer perceptron (MLP) was trained on the training set, with hyperparameters selected on the internal validation set. The structure of the MLP used for tile-level classification is shown in Table S3. The loss function was cross-entropy, and model optimization was performed using Adam with a learning rate of 0.0001. The batch size was 512, the number of training epochs was 500, and the dropout rate was 0.2. An early stopping strategy based on validation loss was applied to prevent overfitting.

Table S3 Network model structure.

| Layer | Type | Number of neurons | Activation |
| --- | --- | --- | --- |
| 1 | Input layer | 1024 or 512 | - |
| 2 | Full connection layer | 256 | ReLU |
| 3 | Dropout layer | - | - |
| 4 | Full connected layer | 32 | ReLU |
| 5 | Output layer | 2 | SoftMax |

To assess the effectiveness of CLAM as a feature extractor, its performance in tile-level pCR classification was compared with two alternative encoders, namely two alternative encoders, namely UNI and CONCH, which represent widely used foundation/contrastive histopathology encoders, respectively.

A single MLP classifier was trained to predict pCR (1/0) using all tiles, and performance was subsequently stratified by tissue compartments (tumor, stroma, iTILs, sTILs, and TILs) based on the classification-derived labels for compartment-specific reporting. The AUC performances for the Tumor, Stroma, iTILs, sTILs, and TILs regions were summarized, and the results for internal and external validation are reported in Table S4 and Table S5, respectively.

Table S4 AUC performance for tile-level pCR classification across five tissue compartments on the Yale Response (internal validation).

| Model | Tumor | Stroma | iTILs | sTILs | TILs |
| --- | --- | --- | --- | --- | --- |
| CLAM | 0.756 | **0.621** | **0.700** | **0.742** | **0.658** |
| UNI [70] | **0.790** | 0.600 | 0.501 | 0.694 | 0.646 |
| CONCH [71] | 0.654 | 0.602 | 0.508 | 0.654 | 0.613 |

Note: The best result in each column is highlighted in **bold**.

Table S5 AUC performance for tile-level pCR classification across five tissue compartments on the IMPRESS HER2+ (external validation) datasets.

| Model | Tumor | Stroma | iTILs | sTILs | TILs |
| --- | --- | --- | --- | --- | --- |
| CLAM | **0.732** | **0.723** | 0.701 | **0.698** | **0.656** |
| UNI [70] | 0.727 | 0.713 | 0.690 | 0.663 | 0.539 |
| CONCH [71] | 0.725 | 0.712 | **0.725** | 0.682 | 0.646 |

Note: The best result in each column is highlighted in **bold**.

As shown in Tables S4 and S5, CLAM achieved the highest or near-highest AUCs in most tissue compartments on both the Yale Response and IMPRESS HER2+ datasets. Its consistent performance across tumor, stromal, and immune-related regions demonstrates superior feature representation and generalization compared with UNI and CONCH, confirming the effectiveness of CLAM as a weakly supervised feature extractor for pCR prediction.

**Supplementary Section S5. Comparison of ROC Curves for pCR Prediction**


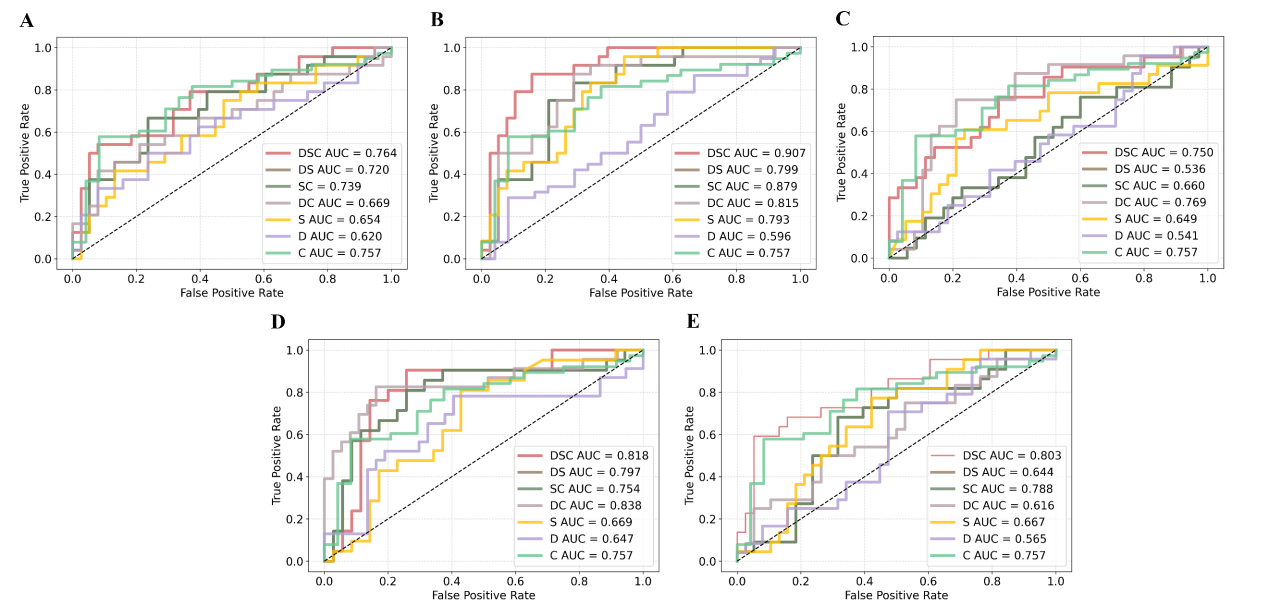


Fig. S2. Tissue-specific ROC curves comparing different feature combinations for pCR prediction. (A–E) correspond to tumor, stroma, iTILs, sTILs, and TILs. Abbreviations: DSC: deep learning-derived pCR scores (DLPS), SNA features, and clinical variables, DS: DLPS and SNA features, SC: SNA features and clinical variables, DC: DLPS and clinical variables, S: SNA features, D: DLPS, C: clinical variables.

**Supplementary Section S6. Relative feature calculation formula**

**Tumor-to-Stroma Ratio (TSR)**

$$\begin{aligned} TSR=\frac{Tumor Tile Counts}{Stroma Tile Counts+Tumor Tile Counts}\#\left( 6 \right) \end{aligned}$$

**iTILs-to-Tumor Ratio (iLTR)**

$$\begin{aligned} iLTR=\frac{iTILs Tile Counts}{Tumor Tile Counts+iTILs Tile Counts}\#\left( 7 \right) \end{aligned}$$

**sTILs Ratio(sTILR)**

$$\begin{aligned} sTILR=\frac{sTILs Tile Counts}{Stroma Tile Counts+sTILs Tile Counts}\#\left( 8 \right) \end{aligned}$$

**TILs-to-Stroma Ratio (TISR)**

$$\begin{aligned} TISR=\frac{TILs Tile Counts}{TILs Tile Counts+Stroma Counts}\#\left( 9 \right) \end{aligned}$$

**Lymphocyte Density (LD)**

$$\begin{aligned} LD=\frac{TILs Tile Counts}{ToTal Tissue Counts}\#\left( 10 \right) \end{aligned}$$

**Supplementary Section S7. DeLong test–based comparison of AUC performance on the IMPRESS HER2+ cohort**

To statistically assess differences in discriminative performance between models, DeLong tests were performed on the IMPRESS HER2+ external validation cohort. For each model, the area under the receiver operating characteristic curve (AUC) and the corresponding 95% confidence interval (CI) were estimated using DeLong’s method. Pairwise DeLong tests were conducted to compare each model against the stromal model, which achieved the highest AUC and was therefore used as the reference. All P values in Table S6 are from pairwise DeLong tests comparing each method against the stromal model as the reference.

Table S6. AUC performance and DeLong test results of the proposed tissue-specific framework on the IMPRESS HER2+ cohort.

| Type | Method | AUC | 95% CI | P value |
| --- | --- | --- | --- | --- |
| Our Method | Tumor (SNA + DLPS + Clinic) | 0.764 | 0.639-0.889 | 0.0219 |
|  | Stroma (SNA + DLPS + Clinic) | **0.907** | 0.833-0.981 | - |
|  | iTILs (DLPS + Clinic) | 0.769 | 0.719-0.957 | 0.047 |
|  | sTILs (DLPS + Clinic) | 0.838 | 0.683-0.922 | 0.044 |
|  | TILs (SNA + DLPS + Clinic) | 0.803 | 0.643-0.894 | 0.040 |
| Clinical | ER | 0.658 | 0.533-0.783 | < 0.001 |
|  | ER% | 0.668 | 0.531-0.805 | 0.003 |
|  | PR | 0.669 | 0.538-0.778 | < 0.001 |
|  | PR% | 0.645 | 0.521-0.770 | < 0.001 |
|  | CEP17 | 0.573 | 0.418-0.728 | < 0.001 |
|  | HER2/CEP17 ratio | 0.674 | 0.521-0.824 | < 0.001 |
| Tiles Count | Tumor | 0.615 | 0.471-0.759 | < 0.001 |
|  | Stroma | 0.680 | 0.543-0.817 | 0.003 |
|  | iTILs | 0.651 | 0.413-0.712 | < 0.001 |
|  | sTILs | 0.651 | 0.512-0.791 | < 0.001 |
|  | TILs | 0.596 | 0.449-0.744 | < 0.001 |
| Relative | TSR | 0.576 | 0.429-0.724 | < 0.001 |
|  | iLTR | 0.562 | 0.409-0.716 | < 0.001 |
|  | sTILR | 0.571 | 0.422-0.720 | < 0.001 |
|  | TISR | 0.587 | 0.420-0.716 | < 0.001 |
|  | LD | 0.518 | 0.462-0.673 | < 0.001 |

Note: The best results for each compartment are bolded in black.

As summarized in Table S6, the stromal model demonstrated the strongest overall performance (AUC = 0.907, 95% CI: 0.833–0.981). While its AUC was numerically higher than those of other tissue-specific models, the DeLong tests indicated modest but statistically significant improvements over several alternative approaches. In contrast, comparisons with clinical-only models, tile-count–based features, and relative spatial metrics consistently showed statistically significant differences, supporting the added value of the proposed tissue-specific framework on the external validation cohort.

**Supplementary** **Section S8. Metrics for percent ablation across tissues**

This table presents the AUC, F1 score, PPV, recall, and NPV for models trained on 20%, 40%, 60%, and 80% subsets of the training data. Each model used a combination of DLPS features, SNA metrics, and clinical variables, and was evaluated on the external IMPRESS HER2+ validation cohort. Results are reported separately for tumor, stroma, iTIL, sTIL, and lymphocyte compartments.

Table S7. Percentage ablation experiments across five tissue compartments on the IMPRESS HER2+ external validation dataset.

| Compartment | Proportions | AUC | F1 score | PPV | Recall | NPV |
| --- | --- | --- | --- | --- | --- | --- |
| Tumor | 0.2 | 0.610 | 0.741 | 0.698 | 0.789 | 0.571 |
|  | 0.4 | 0.617 | 0.718 | 0.700 | 0.737 | 0.542 |
|  | 0.6 | 0.661 | 0.771 | 0.711 | 0.842 | 0.632 |
|  | 0.8 | 0.700 | 0.786 | 0.717 | 0.868 | 0.667 |
|  | 1.0 | 0.764 | 0.828 | 0.735 | 0.947 | 0.800 |
| Stroma | 0.2 | 0.700 | 0.784 | 0.644 | **1.000** | 0.800 |
|  | 0.4 | 0.739 | 0.780 | 0.727 | 0.842 | 0.650 |
|  | 0.6 | 0.787 | 0.809 | 0.706 | 0.947 | 0.769 |
|  | 0.8 | 0.855 | 0.843 | 0.778 | 0.921 | 0.789 |
|  | 1.0 | **0.907** | **0.872** | **0.850** | 0.895 | 0.792 |
| iTILs | 0.2 | 0.569 | 0.765 | 0.674 | 0.886 | 0.583 |
|  | 0.4 | 0.610 | 0.782 | 0.654 | 0.971 | 0.667 |
|  | 0.6 | 0.630 | 0.725 | 0.644 | 0.829 | 0.462 |
|  | 0.8 | 0.673 | 0.819 | 0.708 | 0.971 | 0.800 |
|  | 1.0 | 0.750 | 0.805 | 0.673 | **1.000** | **0.833** |
| sTILs | 0.2 | 0.586 | 0.744 | 0.674 | 0.829 | 0.533 |
|  | 0.4 | 0.682 | 0.775 | 0.689 | 0.886 | 0.615 |
|  | 0.6 | 0.701 | 0.721 | 0.846 | 0.629 | 0.562 |
|  | 0.8 | 0.784 | 0.838 | 0.795 | 0.886 | 0.737 |
|  | 1.0 | 0.818 | 0.833 | 0.811 | 0.857 | 0.714 |
| TILs | 0.2 | 0.554 | 0.792 | 0.655 | **1.000** | 0.750 |
|  | 0.4 | 0.609 | 0.727 | 0.718 | 0.737 | 0.522 |
|  | 0.6 | 0.663 | 0.747 | 0.757 | 0.737 | 0.560 |
|  | 0.8 | 0.738 | 0.763 | 0.763 | 0.763 | 0.583 |
|  | 1.0 | 0.803 | 0.857 | 0.783 | 0.947 | 0.812 |

Note: The best result in each column is highlighted in **bold**.
